# Important Food Sources of Fructose‐Containing Sugars and Incident Hypertension: A Systematic Review and Dose‐Response Meta‐Analysis of Prospective Cohort Studies

**DOI:** 10.1161/JAHA.118.010977

**Published:** 2019-12-12

**Authors:** Qi Liu, Sabrina Ayoub‐Charette, Tauseef Ahmad Khan, Fei Au‐Yeung, Sonia Blanco Mejia, Russell J. de Souza, Thomas M.S. Wolever, Lawrence A. Leiter, Cyril W.C. Kendall, John L. Sievenpiper

**Affiliations:** ^1^ Toronto 3D Knowledge Synthesis and Clinical Trials Unit Clinical Nutrition and Risk Factor Modification Centre St. Michael's Hospital Toronto Ontario Canada; ^2^ Department of Nutritional Sciences Faculty of Medicine University of Toronto Toronto Ontario Canada; ^3^ Division of Endocrinology and Metabolism St. Michael's Hospital Toronto Ontario Canada; ^4^ Department of Health Research Methods, Evidence, and Impact Faculty of Health Sciences McMaster University Hamilton Ontario Canada; ^5^ Li Ka Shing Knowledge Institute St. Michael's Hospital Toronto Ontario Canada; ^6^ College of Pharmacy and Nutrition University of Saskatchewan Saskatoon Saskatchewan Canada

**Keywords:** dairy, fruit, fruit juice, hypertension, SSBs, yogurt, Epidemiology, Hypertension, Diet and Nutrition

## Abstract

**Background:**

Sugar‐sweetened beverages are associated with hypertension. We assessed the relation of important food sources of fructose‐containing sugars with incident hypertension using a systematic review and meta‐analysis of prospective cohort studies.

**Methods and Results:**

We searched MEDLINE, EMBASE, and Cochrane (through December week 2, 2018) for eligible studies. For each food source, natural log‐transformed risk ratios (RRs) for incident hypertension were pooled using pair‐wise meta‐analysis and linear and nonlinear dose‐response meta‐analyses. Certainty in our evidence was assessed using Grading of Recommendations Assessment, Development and Evaluation. We identified 26 reports, including 15 prospective cohorts (930 677 participants; 363 459 cases). Sugar‐sweetened beverages showed harmful (RR_per‐355‐mL_, 1.10 [95% CI, 1.08, 1.12]) whereas fruit (RR_per‐240‐g_, 0.94 [95% CI, 0.96, 0.99]) and yogurt showed protective associations (RR_per‐125‐g_, 0.95 [95% CI, 0.94, 0.97]) with incident hypertension throughout the dose range. One hundred percent fruit juice showed a protective association only at moderate doses (RR_at‐100‐mL_, 0.97 [95% CI, 0.94, 0.99]). The pair‐wise protective association of dairy desserts was not supported by linear dose‐response analysis. Fruit drinks or sweet snacks were not associated with hypertension. Certainty of the evidence was “low” for sugar‐sweetened beverages, 100% fruit juice, fruit, and yogurt and “very low” for fruit drinks, sweet snacks, and dairy desserts.

**Conclusions:**

The harmful association between sugar‐sweetened beverages and hypertension does not extend to other important food sources of fructose‐containing sugars. Further research is needed to improve our estimates and better understand the dose‐response relationship between food sources of fructose‐containing sugars and hypertension.

**Registration:**

URL: https://www.clinicaltrials.gov/. Unique identifier: NCT02702375.

**Clinical Perspective**

**What Is New?**

- Fructose intake is purported to elevate blood pressure.
- Dietary guidelines and public health policy are moving from nutrient‐ to food‐ and dietary pattern–based recommendations.
- We examined the relation of important food sources of fructose‐containing sugars with incident hypertension.

**What Are the Clinical Implications?**

- We identified the following associations of food intake with incident hypertension: harmful: sugar‐sweetened beverages; protective: fruit, yogurt, and 100% fruit juice (moderate dose only); and no association: dairy desserts, fruit drinks, and sweet snacks.
- Overall, this systematic review and meta‐analysis of 26 reports, including 15 unique prospective cohorts, showed that only sugar‐sweetened beverages as a food source of fructose‐containing sugars have a harmful association with incident hypertension.

## Introduction

Hypertension is a major risk factor for developing cardiovascular disease (coronary heart disease and stroke).^1^ The global prevalence of hypertension has been increasing in the past decades.^2^ The World Health Organization attributes the increasing prevalence of hypertension to certain individual behavioural risk factors, including unhealthy dietary choices.^2^ Fructose and fructose‐containing sugars have been implicated as a dietary contributor to the development of hypertension.^3^, ^4^, ^5^ The suggested mechanism is thought to involve uric acid, whereby high intakes of fructose raise uric acid, which, in turn, activates the renin‐angiotensin system and inhibits the nitric oxide system, leading to hypertension.^4^, ^5^, ^6^

Sugar‐sweetened beverages (SSBs) are a major source of fructose in the North American diet.^7^ Although systematic reviews and meta‐analyses of prospective cohort studies have shown a consistent association between SSBs and incident hypertension,^8^ the same has not been shown for the fructose‐containing sugars they contain independent of food form, both in prospective cohort studies and in controlled feeding trials.^9^, ^10^ It is also unclear whether the association observed for SSBs holds for other important food sources of fructose‐containing sugars, such as fruit and fruit‐based products, grains and grain‐based products, dairy and dairy‐based products, and sweets and desserts. As dietary guidelines and public health policy move from nutrient‐based recommendations toward food‐ and dietary pattern–based recommendations,^11^, ^12^, ^13^ it is important to understand the contribution of these different food sources of sugars to the risk of hypertension. To address this gap, we conducted a systematic review and meta‐analysis of prospective cohort studies of the relation of important food sources of fructose‐containing sugars and incident hypertension.

## Methods

The authors declare that the methods have been made publicly available with the registered study protocol (ClinicalTrials.gov; identifier, NCT02702375), and that all supporting data are available within the article and the online Supporting Information.

### Design

We followed the *Cochrane Handbook for Systematic Reviews of Interventions* 14 for the conduct of our systematic review and meta‐analysis and reported our results according to the MOOSE (Meta‐Analysis of Observational Studies in Epidemiology) and Preferred Reporting Items for Systematic Reviews and Meta‐Analyses (PRISMA) guidelines.^15^, ^16^

### Search Strategy

We conducted systematic searches in MEDLINE, EMBASE, and Cochrane Library databases through December 13, 2018 with no language restriction (Table S1). Targeted manual searches served to supplement database searches; these included finding related articles from references of review articles, perusing articles with data from major prospective cohorts that usually report dietary data and speaking to experts in the field. Our search terms reflect the most‐consumed food sources of fructose‐containing sugars in the North American diet^17^, ^18^ (eg, “fructose,” “sugar‐sweetened beverage,” “fruit,” “yogurt,” “ice cream,” and “sweets”) as well as our study design (eg, “prospective study”) and outcome of interest (eg, “hypertension”).

### Study Selection

We included all prospective cohort studies of ≥1 year duration that assessed the association of important food sources of fructose‐containing sugars, including nonalcoholic beverages (eg, SSBs), grain and grain‐based products, fruit and fruit‐based products, dairy and dairy‐based products, and sweets and desserts with incident hypertension in participants free of hypertension at the start of the study. If several studies provided results on the same outcome and used overlapping groups of individuals, we included the study with the longest follow‐up. Abstracts and unpublished studies were not included.

### Data Extraction

Two independent reviewers (Q.L., S.A.C.) extracted relevant data using a standardized proforma. The main outcome was incident hypertension expressed as risk ratios (RRs) with 95% CIs. Data on the amount of food source consumption, distribution of cases and person‐years, and RRs and 95% CIs were extracted. Translation of articles published in languages other than English was done online or by colleagues fluent in the languages. Disagreements were reconciled by consensus. Authors were contacted for missing data.

### Risk of Bias

The same 2 independent reviewers (Q.L., S.A.C.) assessed each study for risk of bias using the Newcastle–Ottawa Scale (NOS) for prospective cohort studies.^19^ NOS points were awarded based on cohort selection, adequacy of outcome measures, and comparability of cohorts regarding design or analysis.^19^ A maximum of 9 points could have been awarded, with 6 points as a minimum threshold for the study to be considered higher quality.^12^ Differences were resolved by consensus.

### Statistical Analyses

Primary pooled pair‐wise analyses were conducted using Review Manager (RevMan 5.3; The Nordic Cochrane Centre, The Cochrane Collaboration, Copenhagen, Denmark), whereas the dose‐response meta‐analyses, subgroup analyses, and publication bias analyses were performed using Stata software (version 15; StataCorp LP, College Station, TX). Each food source of fructose‐containing sugar was considered as an exposure with incident hypertension as the outcome. We used the RR results from multivariable models with the most complete adjustment for potential confounders. Reported odds ratios and hazard ratios were considered an approximation of the RR.^20^ We used natural log‐transformed RRs and 95% CIs for all the analysis and reported results back in the original scale as RRs and 95% CIs. We used 3 separate meta‐analysis methods to assess the association of each food source with hypertension.

We performed: (1) a pair‐wise meta‐analysis comparing highest‐ versus the lowest‐dose categories separately for each food source of fructose‐containing sugars using the DerSimonian–Laird random‐effects model.^21^ We used a fixed‐effects model if the number of studies was ≤5.^22^

We performed (2) a fixed‐effects dose‐response meta‐analysis to estimate linear and (3) nonlinear dose‐relationships using the method of Greenland and Longnecker^23^, ^24^ as described by Orsini^25^, ^26^ and Crippa et al.^27^ In this method, the RRs across all the dose categories of food sources and their 95% CIs are used to estimate the study‐specific slope lines and combined to obtain an overall average slope, taking into account the correlation between summary estimates. The reason for using fixed effects was to minimize the undue influence of exaggerated results from extreme categories on the resulting study‐specific slopes,^27^ to calculate an estimate of heterogeneity using the equivalent 2‐stage method, and to provide robust overall average estimates for the dose‐response association without additional assumptions.^28^

For this analysis, dose was standardized to the same unit for each food source. If consumption was reported by servings per period of time, we converted it into grams or milliliters per day. We defined the assigned dose as the mean consumption in each category of food source. If the assigned doses were not reported, we approximated the mean dose for each category by using the midpoint of its lower and upper bounds. If the lowest‐dose category of a study was open ended, we defined the lowest dose as 0. For open‐ended upper categories, we took half of the adjacent category range to estimate the assigned dose. When cohort size or person‐year per category was not available, categories were regarded equal in size, and follow‐up and the case number per category was obtained by Bekkering's method.^29^ For the nonlinear dose‐response analysis, we fitted the model using restricted cubic splines with 3 knots at the 15th, 50th, and 85th distribution percentiles. If restricted cubic splines could not be calculated because of a limited number of observations, we fitted a second‐order fractional polynomial curve to the data^26^ and tested for goodness of fit of the model using the Akaike information criteria, deviance test (D), and the coefficient of determination (*R* 2) to select the best‐fitting model.^30^ We reported nonlinear associations as the main result for a study if the Wald test for departure from linearity was significant at *P*<0.10. RRs below 1 were considered as protective and above 1 as harmful associations.

For all 3 methods, interstudy heterogeneity was assessed using the Cochran *Q* (χ^2^) statistic and quantified by the I^2^ statistic, where I^2^≥50% and P_Q_<0.1 represented evidence of substantial heterogeneity.^31^, ^32^ For dose‐response meta‐analyses, the I^2^ and Cochrane *Q* statistics were estimated using the 2‐stage method,^33^ and, given that the P_Q_ had excessive power because of too many comparisons,^32^ we multiplied it by the number of comparisons to equalize it with the P_Q_ from a pair‐wise meta‐analysis.

For the pair‐wise meta‐analysis, we explored sources of heterogeneity by sensitivity and subgroup analyses. Sensitivity analysis, in which each study was systematically removed, was carried out to explore the impact of individual studies on the pooled association estimates for each food source. If ≥10 cohort comparisons were available,^14^ then a priori subgroup analyses were performed by meta‐regression for follow‐up (<10 years versus ≥10 years), sex (males versus females versus mixed), study quality (NOS<6 versus ≥6), age (<median versus ≥median), and funding source (agency versus industry versus mixed). As part of the sensitivity analysis, we also performed a pooled analysis of primary studies using extreme comparisons. If ≥10 cohort comparisons were available, then publication bias was assessed by visual inspection of funnel plot and statistical evaluation using the the Begg^34^ and Egger^35^ tests, with significance set at *P*<0.10. In the presence of publication bias, we used the Duval and Tweedie trim‐and‐fill method to adjust for funnel‐plot asymmetry by imputing missing study data.^36^

The STATA code for SSBs dose‐response analysis is provided in Data S1, and dose‐response raw data are provided in Data S2.

### Grading of the Evidence

Overall quality and strength of the evidence at was assessed using the GRADE (Grading of Recommendations Assessment, Development and Evaluation) approach.^37^ Our certainty in the evidence was graded as “high,” “moderate,” “low,” or “very low.” Observational studies receive an initial grade of “low” and then can be down‐ or upgraded based on prespecified criteria. Criteria to downgrade included risk of bias (weight of studies show risk of bias as assessed by NOS<6), inconsistency (substantial unexplained interstudy heterogeneity, I^2^>50%; *P*<0.10), indirectness (presence of factors that limit the generalizability of the results), imprecision in the pooled‐risk estimate (the 95% CI for risk estimates are wide or cross a minimally important difference of 10% for protection or harm [RR, 0.9–1.1]), and publication bias (evidence of small‐study effects).^37^ In contrast, criteria to upgrade included a large magnitude of effect (RR>2 or RR<0.5 in the absence of plausible confounders), dose‐response gradient, and attenuation of the pooled‐effect estimate by plausible confounders.^37^

## Results

### Search Results

**Figure 1:**
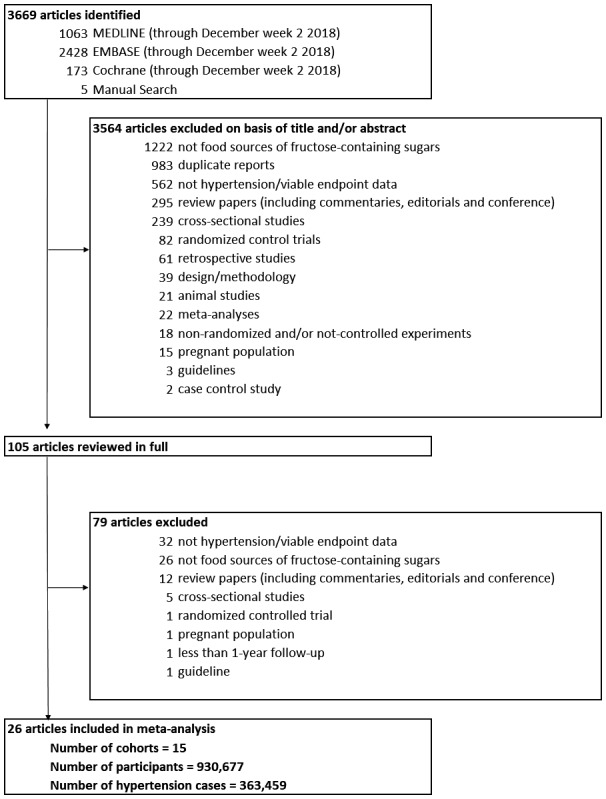
Flow of the literature search.

Figure 1 shows the flow of the literature search. Of 3669 reports, 26 reports^38^, ^39^, ^40^, ^41^, ^42^, ^43^, ^44^, ^45^, ^46^, ^47^, ^48^, ^49^, ^50^, ^51^, ^52^, ^53^, ^54^, ^55^, ^56^, ^57^, ^58^, ^59^, ^60^, ^61^, ^62^, ^63^ with data from 15 unique prospective cohort studies met our inclusion criteria involving a total of 930 667 participants with 363 459 incident cases of hypertension. There were 13 cohort comparisons (427 630 participants [n]; 120 553 cases) for SSBs; 13 cohort comparisons (n=281 120; 148 928 cases) for fruit, 1 of which was from a case‐cohort report; 9 cohort comparisons (n=235 705; 97 783 cases) for yogurt; 3 cohort comparisons (n=41 398; 12 106 cases) for dairy desserts; 2 cohort comparisons for 100% fruit juice (n=83 178; 46 811 cases); and 1 cohort comparison each for fruit drinks (n=424; 47 cases) and sweet snacks (n=439; 45 cases). Definitions of the food categories, as defined by the cohort studies, can be found in Table S2. We assumed that yogurt was a source of fructose, given that consumers prefer yogurt products with a moderate (≈7–10%) concentration of added sucrose.^64^, ^65^, ^66^ We did not identify prospective cohort studies assessing the relation of grain and grain‐based products or other fruit‐ or dairy‐based products with incident hypertension. Two studies sent additional data that we could use.^56^, ^57^

### Study Characteristics

Table shows the characteristics of the included prospective cohort studies. Participants were from 7 countries, the majority from the United States, with a median age of 44 (range, 14–65) years. One cohort was conducted in children and teens (age range, 6–18 years),^43^, ^53^ 1 in young adults (age range, 18–30 years),^42^, ^47^ and the remaining 13 cohorts studies in general samples of adults. Median follow‐up periods were 10 years (range, 3.6–28.0) for SSBs; 9 years (range, 4–26) for fruit; 14.6 years (range, 5–30) for yogurt; 10 years (range, 9–15) for dairy desserts; and 13.9 years (range, 7.8–20.0) for 100% fruit juice; and the follow‐up period was 3.6 years for both fruit drinks and sweet snacks. Dietary intake assessments were performed with validated food frequency questionnaires in all studies. Intakes (rounded to the nearest 5) for SSBs, fruit, yogurt, dairy desserts, 100% fruit juice, fruit drinks, and sweet snacks ranged from 0 to 1420 mL/d, 0 to 640 g/d, 0 to 320 g/d, 5 to 530 mL/d, 0 to 230 mL/d, 0 to 70 mL/d, and 5 to 75 g/d, respectively. Ascertainment of incident cases of hypertension was done by independent blind assessment in 7 cohort studies^38^, ^39^, ^40^, ^42^, ^44^, ^46^, ^47^, ^48^, ^49^, ^50^, ^52^, ^54^, ^55^ and by self‐report in the other 8 cohort studies.^41^, ^43^, ^45^, ^48^, ^51^, ^53^, ^56^, ^57^, ^58^, ^59^, ^60^, ^61^, ^62^ All cohort studies defined individuals with hypertension to have elevated systolic and/or diastolic blood pressure (BP) or take antihypertensive medication. The systolic BP cutoff ranged from 130 to 140 mm Hg, whereas the diastolic BP cutoff ranged from 80 to 90 mm Hg. All reports were funded by agency alone, except 3 reports^50^, ^51^, ^55^ which were funded by both agency and industry.

**Table 1:** Cohort Characteristics

Study, Year (reference)	Cohort	Population at Baseline	Country	Participants	Incident Cases	Age (y)	Years Range	Duration (mean or median)	Dietary Intake Assessment	Frequency of Administration	Quantile Division	Exposure (median or range)^a^	Serving Size	Outcome Assessment	Funding Sources
Sugar‐sweetened beverages (SSBs)															
Barrio‐Lopez et al, 2013^38^	SUN	Nonhypertensive; does not meet any of the criteria for MetS; not abnormal energy intake^b^	Spain	8157	1464	36 (mean)	2004 to 2012	6 y	Validated FFQ	Every 2 y	Quintile	(change in consumption) −1.35 to 2.4 servings/wk	330 mL	Validated self‐report	Agency
Cohen et al, 2012^40^	NHS	Nonhypertensive	US	88 540 (F)	42 022	38 to 53	1980 to 2008	28 y	Validated FFQ	Every 4 y	Quartile	<1/mo to ≥1/d	Bottle, glass, or can	Self‐reported Physician diagnosed	Agency
	NHSII			97 991 (F)	21 873	31 to 40	1991 to 2007	16 y							
	HPFS			37 360 (M)	13 439	42 to 63	1986 to 2008	22 y							
Dhingra et al, 2007^41^	FOC	Nonhypertensive; no baseline MetS, no prevalent CVD	US	2803	1377	53 (mean)	1987 to 2001	4 y	Validated FFQ	Years 0,4	Quartile	0 to ≥2 servings/d	12 oz	Independent blind assessment	Agency
Duffey et al, 2010^42^	CARDIA	Nonhypertensive; no baseline MetS	US	2639	609	18 to 30	1986 to 2006	20 y	Validated SFFQ	Years 0,7	Quartile	n/a	n/a	Self‐reported Physician diagnosed	Agency
Kang et al, 2017^58^	KoGES	Nonhypertensive; no baseline MetS	Korea	4591	1309	40 to –69	(2001–2002) to (2009–2010)	10 y	Validated SFFQ	Every 2 y	Quartile	0 to ≥4 servings/wk	250 mL	Independent blind assessment	Agency
Kwak et al, 2018^59^	KoGES	Nonhypertensive; no CVD; no diabetes mellitus; no cancer	Korea	5775	1175	40 to 69	(2001–2002) to (2009–2010)	10 y	Validated SFFQ	Every 2 y	Quartile	0 to 3.5 servings/wk	n/a	Independent blind assessment	Agency
Mirmiran et al, 2015^43^	TLGS	Nonhypertensive; within ±3 SD of energy intake	Iran	424	47^c^	14 (mean)	(2006–2008) to (2009–2011)	3.6 y	Validated SFFQ	Every 3 y	Quartile	1.12 to 100 mL/d	250 mL	Independent blind assessment	Agency
Sayon‐Orea et al, 2015^46^	SUN	Nonhypertensive; not abnormal energy intake^b^; no chronic disease (cancer, diabetes mellitus, or CVD)	Spain	13 843	1308	36 (mean)	1999 to 2010	8.1 y	Validated SFFQ	Years 0,6	Tertile	0 to ≥7 servings/wk	6.7 oz	Validated self‐report	Agency
Weng et al, 2013^60^	ARIC	Nonhypertensive; no abnormal energy intake^d^	US	9913	2853	45 to 64	(1987–1989) to (1996–1998)	9 y	Validated FFQ	Years 0,3	Tertile	0 to ≥1 serving/d	n/a	Independent blind assessment	Agency
Winkelmayer et al, 2005^52^	NHS	Nonhypertensive	US	61 091 (F)	19 541	30 to 55	1990 to 2002	12 y	Validated FFQ	Every 4 y	Quartile	<1 serving/d to ≥4/d	Serving size as indicated on FFQ	Self‐reported Physician diagnosed	Agency
	NHSII			94 503 (F)	13 536	25 to 42	1991 to 2003					<1 serving/d to (4–5)/d			
Fruit															
Auerbach et al, 2017^54^	WHI	Nonhypertensive; not abnormal energy intake^e^	US	80 539 (F)	46 202	50 to 79	(1993–1998) to (2004 to 2005)	7.8 y	Validated SFFQ	Every 6 to 12 mo	Quintile	0.3 to 2.4 servings/d	n/a	Self‐reported Physician diagnosed	Agency
Borgi et al, 2016^39^	NHS	Nonhypertensive	US	39 164 (F)	35 375	30 to 55	1984 to 010	26 y	Validated FFQ	1984, 1986, every 4 y after	Quintile	≤4 servings/wk to ≥4 servings/d	Dependent on type of fruit^f^	Self‐reported Physician diagnosed	Agency
	NHSII			63 885 (F)	25 246	25 to 42	1991 to 2011	20 y							
	HPFS			20 010 (M)	16 752	40 to 75	1986 to 2010	24 y							
Kim et al, J Acad Nutr Diet, 2017^63^	KoGES	Nonhypertensive; no CVD; no cancer; no abnormal energy intake^g^	Korea	2005 (M)	606	40 to 69	(2001–2002) to (2009–2010)	8 y	Validated SFFQ	Every 2 y	Quartile	0 to ≥4 servings/d	100 g	Independent blind assessment	Agency
				2174 (F)	552										
Koochakpoor et al, 2018^62^	TLGS (case‐cohort analysis)	Nonhypertensive; no MetS at baseline; no CVD	Iran	640 cases 644 controls		42	2002 to 2014	12 y	Validated SFFQ	Every 3 y	Quartile	n/a	n/a	Independent blind assessment	Agency
Nunez‐Cordoba et al, 2009^44^	SUN	Nonhypertensive; no CVD; not abnormal energy intake^h^	Spain	8594	426	20 to 95	1999 to 2006	4.1 y	Validated SFFQ	Every 2 y	Quintile	≤1 to >4 servings/d	Serving size as indicated on FFQ	Validated self‐report	Agency
Psaltopoulou et al, 2004^45^	EPIC	Nonhypertensive	Greece	20 343	5424	20 to 86	1994 to 1999	5 y	Validated SFFQ	Every 3 to 5 y	Per SD increment	<1 to >3 servings/d	n/a	Independent blind assessment	Agency
Steffen et al, 2005^47^	CARDIA	Nonhypertensive; not abnormal energy intake^i^; no diabetes mellitus	US	4304	997	18 to 30	1986 to 2001	15 y	Validated SFFQ	Years 0,7	Quintile	<0.2 to >1.5 times/d	Frequency not servings	Independent blind assessment	Agency
Tsubota‐Utsugi et al, 2011^48^	Ohasama	Nonhypertensive; within ±3 SD of energy intake	Japan	745	222	≥35	1998 to 2002	4 y	Validated FFQ	1 (baseline)	Quartile	≤38.40 to ≥100.03 g/d	n/a	Self‐reported	Agency
Wang et al, 2012^50^	WHS	Nonhypertensive; no cancer; no CVD	US	28 082 (F)	13 633	39 to 89	(1992–1995) to 2007	12.9 y	Validated FFQ	1 (baseline)	Quintile	<0.5 to ≥3 servings/d	n/a	Self‐reported Physician diagnosed	Agency and Industry
Weng et al, 2013^60^	ARIC	Nonhypertensive; no abnormal energy intake^d^	US	9913	2853	45 to 64	(1987–1989) to (1996–1998)	9 y	Validated FFQ	Years 0,3	Quintile	n/a	n/a	Independent blind assessment	Agency
Yogurt															
Alonso et al, 2009^56^	ARIC	Nonhypertensive; no CVD; no diabetes mellitus; no abnormal energy intake^d^	US	8208	2399	45 to 64	(1987–1989) to (1996–1998)	9 y	Validated FFQ	Every 3 y	Tertile	0.01 to 1.3 servings/d	n/a	Independent blind assessment	Agency
Buendia et al, 2018^55^	NHS	Nonhypertensive; no CVD; no diabetes mellitus; no cancer; not abnormal energy intake^j^; no abnormal dairy intake^k^	US	69 298	41 934	45 (mean)	1980 to 2010	30 y	Validated SFFQ	Every 4 y	Quintile	<1 serving/mo to ≥5 servings/wk	1 cup	Self‐reported Physician diagnosed	Agency and industry
	NHSII			84 368	26 282	36 (mean)	1989 to 2009	20 y							
	HPFS			30 512	14 166	51 (mean)	1986 to 2010	24 y							
Engberink et al, 2009^57^	MORGEN	Nonhypertensive	Netherlands	3454	713	20 to 65	(1993–1997) to (1998–2002)	5 y	Validated SFFQ	1 (baselines)	Quartile	12 to 122 g/d	n/a	Independent blind assessment	Agency
Kim et al, Brit J Nutr, 2017^61^	KoGES	Nonhypertensive; no MetS at baseline; no CVD; no cancer	Korea	4335	1556	40 to 69	(2001–2002) to (2009–2010)	10 y	Validated SFFQ	Years 0,4	Quartile	0 to ≥4 servings/wk	140 mL	Independent blind assessment	Agency
Steffen et al, 2005^47^	CARDIA	Nonhypertensive; not abnormal energy intake^i^; no diabetes mellitus	US	4304	997	18 to 30	1986 to 2001	15 y	Validated SFFQ	Years 0,7	Tertile	<0.1 to >0.5 times/wk	Frequency not servings	Independent blind assessment	Agency
Wang et al, 2008^49^	WHS	Nonhypertensive, no cancer, no CVD, not “implausible” energy intake	US	28 886 (F)	8710	54 (mean)	(1992–1995) to 2005	10 y	Validated SFFQ	1 (baseline)	Quintile	<1 serving/mo to ≥1 servings/d	Serving size as indicated on SFFQ	Self‐reported Physician diagnosed	Agency
Wang et al, 2015^51^	FHS	Nonhypertensive	US	2340	1026	52 (mean)	1991 to 2008	14.6 y	Validated FFQ	At each exam^l^	Per 1 serving/wk increment	0 to 4.000 servings/wk	227 g	Independent blind assessment	Agency and industry
Dairy desserts															
Alonso et al, 2009^56^	ARIC	Nonhypertensive; no CVD; no diabetes mellitus; no abnormal energy intake^d^	US	8208	2399	45 to 64	(1987–1989) to (1996–1998)	9 y	Validated FFQ	Every 3 y	Tertile	0.04 to 1.5 servings/d	n/a	Independent blind assessment	Agency
Steffen et al, 2005^47^	CARDIA	Nonhypertensive; not abnormal energy intake i; no diabetes mellitus	US	4304	997	18 to 30	1986 to 2001	15 y	Validated SFFQ	Years 0,7	Quintile	<0.1 to >2.2 times/wk	Frequency not servings	Independent blind assessment	Agency
Wang et al, 2008^49^	WHS	Nonhypertensive, no cancer, no CVD, not “implausible” energy intake	US	28 886 (F)	8710	54 (mean)	(1992–1995) to 2005	10 y	Validated SFFQ	1 (baseline)	Quintile	<1 serving/mo to ≥1 servings/d	Serving size as indicated on SFFQ	Self‐reported Physician Diagnosed	Agency
100% fruit juice															
Auerbach et al, 2017^54^	WHI	Nonhypertensive; not abnormal energy intake^e^	US	80 539 (F)	46 202	50 to 79	(1993–1998) to (2004–2005)	7.8 y	Validated SFFQ	Every 6 to 12 mo	Quintile	0 to 7.8 oz/d	100 oz	Self‐reported Physician diagnosed	Agency
Duffey et al, 2010^42^	CARDIA	Nonhypertensive; no baseline MetS	US	2639	609	18 to 30	1986 to 2006	20 y	Validated SFFQ	Years 0,7	Quartile	n/a	8 oz	Self‐reported Physician diagnosed	Agency
Fruit drinks															
Mirmiran et al, 2015^43^	TLGS	Nonhypertensive; not ±3 SD of energy intake	Iran	424	47^c^	14 (mean)	(2006–2008) to (2009–2011)	3.6 y	Validated SFFQ	Every 3 y	Quartile	1.12 to 100 mL/d	250 mL	Independent blind assessment	Agency
Sweet snacks															
Asghari et al, 2016^53^	TLGS	Nonhypertensive; not ±3 SD of energy intake	Iran	439	45^c^	14 (mean)	(2006–2008) to (2009–2011)	3.6 y	Validated SFFQ	Every 3 y	Quartile	7 to 72.8 g/d	n/a	Independent blind assessment	Agency

CVD indicates cardiovascular disease; FFQ, food frequency questionnaire; HDL, high‐density lipoprotein; MetS, metabolic syndrome; n/a, not applicable; SFFQ, semiquantitative food frequency questionnaire; SSBs, sugar‐sweetened beverages.

a There was some variability in how the cohorts chose to represent their exposure levels, such as quantiles used and the frequency of intake vs servings/d intake. We compared the highest to lowest exposure quantile for each cohort study, regardless of the number of qunatiles (tertile, quartile, or quintile). For dose‐response analysis, SSBs and fruit exposure levels were converted to servings. For SSBs, we performed the conversion based on serving sizes indicated in the articles. For fruit, we assumed that 1 serving=1/2 cup=87.5 g=1 instance of intake (frequency).

b Defined as <800 kcal/d in men and <500 kcal/d in women, or >4000 kcal/d in men and >3500 kcal/d in women.

c Study only reported cases of metabolic syndrome, defined as having ≥3 of the following: abdominal obesity, high fasting glucose, low HDL cholesterol, hypertension, or high triglycerides.

d Defined as <700 kcal/d in men and <500 kcal/d in women, or >4500 kcal/d in men and >3500 kcal/d in women.

e Defined as ≤600 kcal/d or ≥5000 kcal/d.

f Serving sizes: raisins (1 oz)/grapes (half cup); apples/pears (1), bananas (1), strawberries (half cup), blueberries (half cup), prunes (half cup), avocado (half), cantaloupe (1/4 melon), oranges (1), peaches/apricots/plums (1 or half cup canned).

g Defined as <500 kcal/d or >6000 kcal/d.

h Defined as <800 kcal/d in men and <500 kcal/d in women, or >4200 kcal/d in men and >3800 kcal/d in women.

i Defined as <800 kcal/d in men and <600 kcal/d in women, or >8000 kcal/d in men and >6000 kcal/d in women.

j Defined as <800 kcal/d for men and <500 kcal/d in women, or >4200 kcal/d in men and >3500 kcal/d in women.

k Defined as ≥6 servings/d of total dairy, >4 servings/d of cheese, or ≥6 servings/d of milk.

l Exams were (1991–1995), (1995–1998), (1998–2001), and (2005–2008).

Table S3 shows the confounding variables included in the most adjusted models for each of the included prospective cohort studies. The median number of variables in the most adjusted models was 12 (range, 7–22). All cohort studies adjusted for the prespecified primary confounding variable (age). Whereas Psaltopoulou et al^45^ only adjusted for 3 of the 8 prespecified secondary confounding variables (smoking, markers of overweight/obesity, energy intake, physical activity, sex, diabetes mellitus, alcohol consumption, and sodium intake), the remaining cohort studies controlled for ≥4.

Table S4 shows the cohort study‐quality assessments by the NOS. Only 2 of the 26 articles included scored <6 on the NOS scale, which denotes lower quality.^50^, ^55^

### Food Sources of Fructose‐Containing Sugars on Incident Hypertension

**Figure 2:**
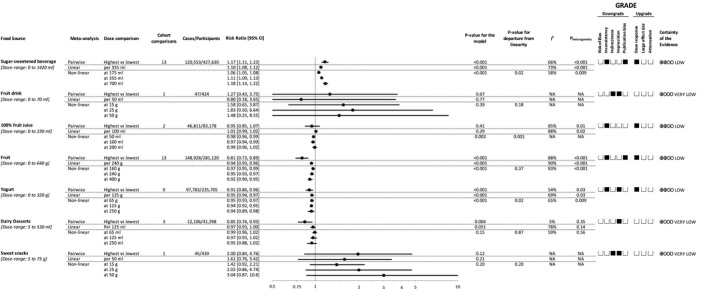
Relation of sources of fructose‐containing sugars and incident hypertension. Pair‐wise summary estimates were derived from pooled risk ratios for highest vs lowest intake of the food sources. Estimates of linear and nonlinear dose‐response relationships are presented per intake level indicated in the column, “dose comparison.” Dose‐ranges are rounded to the nearest five. Data are expressed as risk ratios (RRs) with 95% CIs. Values of I^2^≥50% indicate substantial heterogeneity. RRs >1.0 indicate a harmful association. The Grading of Recommendations, Assessment, Development and Evaluation of prospective cohort studies are rated as “low” certainty of evidence and can be downgraded by 5 domains and upgraded by 3 domains. Filled black squares indicate downgrade or upgrades for each outcome. NA indicates not applicable.

Figure 2 shows the superplot of the summary estimates for pair‐wise, linear, and nonlinear meta‐analyses of the relation of each important food sources of fructose‐containing sugars with incident hypertension.

Figures S1 through S7 show the individual forest plots for the pair‐wise meta‐analysis of highest versus lowest category of intake for the individual food sources of fructose‐containing sugars. Comparing highest versus lowest categories of intake, a harmful association with incident hypertension was shown for SSBs (RR=1.17 [95% CI, 1.11, 1.23]; Figure S1), whereas protective associations were shown for fruit (RR=0.81 [95% CI, 0.73, 0.89]; Figure S2), yogurt (RR=0.91 [95% CI, 0.86, 0.96]; Figure S3), and dairy desserts (RR=0.85 [95% CI, 0.76, 0.95]; Figure S4). Comparing highest versus lowest categories of intake, 100% fruit juice (RR=0.95 [95% CI, 0.85, 1.07]; Figure S5), fruit drinks (RR=1.27 [95% CI, 0.43, 3.75]; Figure S6), or sweet snacks (RR=2.00 [95% CI, 0.84, 4.76]; Figure S7) did not show any association with incident hypertension.

**Figure 3:**
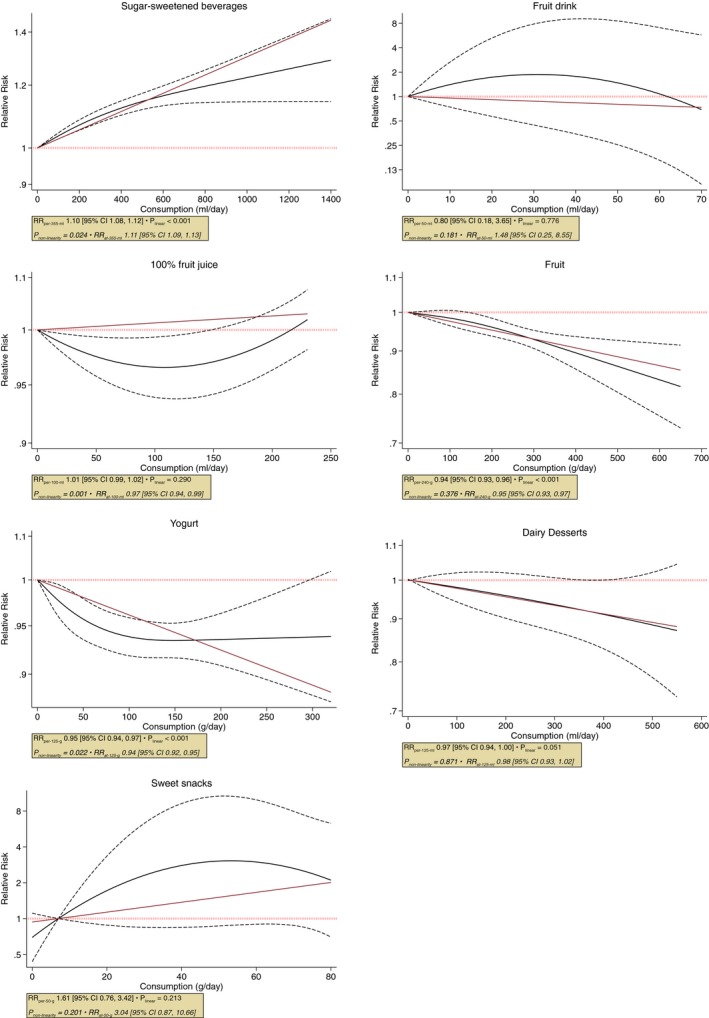
Dose‐response relation between sources of fructose‐containing sugars and incident hypertension. Dose‐response relationship between intake of SSBs, fruit, 100% fruit juice, yogurt, fruit drink, dairy desserts, and sweet snacks with risk of hypertension. Red line represents the linear, and black lines represent the nonlinear models, respectively. Dotted lines represent 95% CIs of the nonlinear model. RR indicates risk ratio; SSBs, sugar‐sweetened beverages.

Figure 2 shows the summary estimates and Figure 3 shows the dose‐response relationships between the individual food source of fructose‐containing sugars and risk of hypertension. Figure S8 has additional study‐specific data points superimposed on the graphs seen in Figure 3.

Using data from 13 cohorts with a dose range of 0 to 1420 mL/d, there was a harmful dose‐response relationship between SSBs intake and hypertension with evidence of nonlinearity (*P* value for departure from linearity=0.02). The nonlinear curve was similar to the linear association with a suggestion of plateauing of risk after 400‐mL/d consumption. The estimated RR at 355 mL (1 serving) of SSBs was 1.11 [95% CI, 1.09, 1.13].

Using data from 2 cohorts with a dose range of 0 to 230 mL/d, there was a nonlinear U‐shaped dose‐response relationship between 100% fruit juice intake and hypertension (*P* value for nonlinearity=0.001). The curve suggested a maximum protective association between 50 and 150 mL/d and appearance of harmful association over intake of 200 mL/day. The estimated RR for 100 mL/d (one‐half serving of small glass) of 100% fruit juice was 0.97 [95% CI, 0.94, 0.99].

Using data from 13 cohorts with a dose range of 0 to 640 g/d, there was a protective linear dose‐response relationship between fruit intake and hypertension (*P* value for departure from linearity=0.46). The estimated RR per 240 g (3 servings) of fruit intake was 0.94 (95% CI, 0.93, 0.96).

Using data from 9 cohorts with a dose range of 0 to 319 g/d, there was a nonlinear protective dose‐response relationship between yogurt intake and hypertension (*P* value for nonlinearity=0.02). The curve suggested a continuous reduction of RR until 100 g/d of intake, followed by a plateau. The estimated RR at 125 g (1 serving) of yogurt was 0.94 (95% CI, 0.92, 0.95).

Using data from 9 cohorts with a dose range of 0 to 530 mL/d, there was no dose‐response relationship between dairy desserts intake and hypertension and no evidence of nonlinearity (*P*=0.87). The estimated RR at 125 mL (1 serving) of dairy dessert was 0.97 (95% CI, 0.93, 1.00), which contrasts against the result from the pair‐wise analysis of highest versus lowest intake.

The associations for SSBs, fruit, yogurt, and 100% fruit juice were all complicated by evidence of substantial heterogeneity (I^2^>50% and P_Q_<0.10) in pair‐wise, linear, and nonlinear analyses, except for 100% fruit juice for which the measure of heterogeneity could not be calculated for nonlinear analysis because of lack of relevant data points.

There were no significant linear or nonlinear dose‐response relationships between fruit drinks or sweet snacks and incident hypertension (Figures 2 and 3 and Figure S8).

### Sensitivity Analyses and Subgroup Analyses

Table S5 shows the recalculation of the association estimates after systematic removal of each cohort study (not available for food groups of ≤2 studies) from the pair‐wise meta‐analysis. Systematic removal of each cohort study for SSBs or fruit did not alter the direction or significance of the association or the evidence of heterogeneity. Systematic removal of each cohort study for yogurt did not alter the direction or significance of the association. However, interstudy heterogeneity of the yogurt food group was altered when Kim et al^61^ was removed from the pooled analysis, where it became nonsignificant (I^2^=30%; *P*=0.19).

Figure S9 shows the subgroup analyses for SSBs, and Figure S10 shows the subgroup analyses for fruit. No subgroup analyses were able to explain the heterogeneity between study estimates in the association of SSBs with hypertension or the association of fruit with hypertension.

### Publication Bias

Figure S11 shows the funnel plot assessing publication bias for SSBs. Visual inspection of the funnel plot showed evidence of asymmetry. Both the Begg (*P*=0.04) and Egger (*P*=0.02) tests indicated evidence of small‐study effects. Adjustment for funnel‐plot asymmetry by the recalculation of the pooled estimate by inputting missing cohort studies using the Duvall and Tweedie trim‐and‐fill method did not alter the significance of the relationship with only limited attenuation of the summary estimate (RR=1.12 [95% CI, 1.05, 1.19] versus original [RR=1.17; 95% CI, 1.11, 1.23]; Figure S12). Figure S13 shows the funnel plot assessing publication bias for fruit. Visual inspection of the funnel plot showed evidence of asymmetry, and the Begg (*P*=0.09) test was significant whereas the Egger (*P*=0.70) test was nonsignificant. The Duvall and Tweedie trim‐and‐fill method did not perform any trimming.

### GRADE Assessment

Table S6 shows a summary of the GRADE assessment. Our certainty in our pooled estimates was “low” for a harmful association for SSBs, protective association at moderate doses for 100% fruit juice, protective association for fruit, and protective association for yogurt; and “very low” for no association for fruit drinks, sweet snacks, and dairy desserts. This was attributable to downgrades for inconsistency (SSBs, 100% fruit juice, fruit, and yogurt), indirectness (fruit drink, sweet snacks), imprecision (fruit drinks, yogurt, dairy desserts, and sweet snacks) and publication bias (SSBs, fruit), and upgrades for dose‐response gradients (SSBs, fruit, yogurt, and 100% fruit juice).

## Discussion

In our systematic review and meta‐analysis, pooled analyses of 26 reports of 15 prospective cohort studies involving 930 677 participants with 363 459 incident cases of hypertension found that SSBs had a harmful association with incident hypertension whereas fruit and yogurt had protective associations with incident hypertension. One hundred percent fruit juice showed a U‐shaped dose‐response association with hypertension, showing protection at moderate doses (100–250 mL). There was no association of fruit drinks, dairy desserts, or sweet snacks with hypertension.

### Findings in the Context of the Literature

Our results are consistent with established research on the harmful association between SSBs and incident hypertension. Our previous systematic review and meta‐analysis found a significant 12% increase in incident hypertension when comparing highest to lowest SSBs intake.^8^ This present study included more studies covering a wider range of cohorts and found a comparable 10% increase in incident hypertension with 1‐serving (355‐mL)/d intake using the linear dose response and 11% increase at 1 serving using the nonlinear dose response. We observed evidence for nonlinearity for SSBs, but the 2 curves (linear and nonlinear) visually suggested high similarity; the difference, though statistically significant, is clinically irrelevant. The dose‐response relationship suggested an increase in risk of hypertension with SSBs intake at all higher doses when compared with no consumption. Other, more‐recent systematic reviews and meta‐analyses have identified a similar association between SSBs intake and incident hypertension.^67^, ^68^ Consistent harmful associations have also been shown with other related cardiometabolic diseases, such as diabetes mellitus, metabolic syndrome, and cardiovascular disease.^69^, ^70^ A possible explanation is that SSBs provide a form of liquid calories that produce less satiety than consumption of solid calories, resulting in overall increased energy intake, weight gain, and downstream hypertension.^71^ Another is that the association between SSBs intake and incident hypertension is confounded by an unhealthy lifestyle.^72^ Though the cohort studies included in our analyses consistently controlled for variables such as energy intake, physical activity, smoking, and alcohol intake, residual confounding could have contributed to the harmful association between SSBs intake and incident hypertension.

We also identified a U‐shaped dose‐dependent relationship between incident hypertension and 100% fruit juice intake, where intake below 200 mL showed protective associations with hypertension. The maximum protective association appeared to be between doses of 50 and 150 mL (≈0.5–1.0 servings), after which the dose‐response curve suggested increasing RR with increasing dose, and even suggested harmful associations over intakes of 200 mL. This is in line with some national health guidelines, in which a 150‐mL glass of fruit juice contributes toward daily fruit consumption.^73^ Other cohort studies have shown that 100% fruit juice, compared with fruit drinks, has neutral^74^ or even protective^75^ associations with incident cardiometabolic disease. The protective association of 100% fruit juice noted at moderate doses may be the result of the range of nutrients and bioactive compounds within the juice.^76^ However, the potential for harmful associations at higher doses may be attributable to the consumption of excess calories outweighing any potentially protective nutrients contained within 100% fruit juice.^77^

We did not find any association of 100% fruit juice intake in the pair‐wise meta‐analysis. This underscores the point that, without consideration of dose‐response relationship, an analysis of extreme intakes ignores the dose entirely, assumes a false‐linear relationship between the lowest and highest intake, and fails to detect important dose ranges for protective or harmful associations. While we argue that highest versus lowest analysis is possibly misleading, we reported it in our article because of our preregistered a priori analysis plan.

Recent systematic reviews and meta‐analyses concur with our results of an inverse dose‐response association between fruit and incident hypertension.^78^, ^79^ We also saw evidence for nonlinearity for fruit. However, similar to the SSBs curve discussed above, the small statistical difference may be clinically irrelevant. The dose‐response relationship suggested a reduction in risk of hypertension with intake of fruit at all increased doses, albeit in the assessed dose‐range when compared with no consumption. Consistent protective associations have been shown for fruit with other related cardiometabolic diseases, such as diabetes mellitus, cardiovascular disease, and all‐cause mortality.^80^, ^81^, ^82^, ^83^ One popular hypothesis of the protective effects of fruit consumption pertains to their high phytochemical, especially flavonoid, content.^84^ These flavonoids have been shown to decrease important factors in the development of hypertension and have been shown to reduce BP.^85^, ^86^, ^87^, ^88^, ^89^, ^90^ Various fruits are also rich in potassium with small amounts of magnesium and calcium, the combination of which has been shown to decrease BP.^91^

We identified a dose‐dependent relationship between incident hypertension and yogurt intake, where intakes between 100 and 250 g/d showed maximum protective associations with hypertension. Our spline analysis of yogurt shows that the risk plateaus after intakes above 100 g/d, and that there is not a sufficient amount of precise data to suggest any more protection associated with increasing intake beyond 250 g/d. Yogurt has shown protective associations with various other cardiometabolic disease outcomes; a large systematic review identified that the consumption of different dairy products (sweetened or not) shows favorable or neutral associations with cardiometabolic outcomes of stroke, cardiovascular disease, coronary artery disease, hypertension, metabolic syndrome, and type 2 diabetes mellitus.^92^ Specific to dairy products that contain fructose, yogurt has shown a protective association with body weight, and both yogurt and ice cream have shown protective associations with diabetes mellitus.^93^, ^94^ The link between dairy and hypertension is unclear. Dairy foods are rich in micronutrients, such as calcium, potassium, and magnesium, which may lower BP by several mechanisms.^95^, ^96^, ^97^ Yogurt contains more calcium, potassium, and magnesium and more protein per serving compared with milk,^98^ and these nutrients may be more bioavailable than in other dairy products.^99^ The probiotics abundant in yogurt have also been found to reduce BP by inhibiting angiotensin‐converting enzyme.^95^ Despite these potential mechanistic explanations, a Mendelian randomization analysis did not find a casual link between dairy intake and reduced incident hypertension in prospective cohorts.^100^

Last, we did not find any associations of dairy desserts, fruit drinks, or sweet snacks with incident hypertension. Although we found a small protective association for dairy desserts when comparing highest versus lowest intake categories, this was not supported by the dose‐response analysis. Dose‐response analysis considers the full dose range and thus is more credible. The contrasting result for dairy desserts again underscores the importance of assessing the dose‐response relationship using all categories rather than just using highest versus lowest analysis, which ignores the differing dose ranges used in different studies. Indeed, the highest category doses in our included studies were 93, 250, and 532 mL/d, a difference of more than twice in each study leading to inaccurate results in the highest versus lowest analysis. An additional limitation of the dairy desserts analysis was that although 2 studies defined dairy desserts as a mix of cakes, ice cream, sherbet, etc,^49^, ^56^ the other study was nonspecific with what the “dairy desserts” category encompassed.^47^

The lack of association for sweet snacks is not surprising, given that the result is only based on one cohort^53^ that examined children and adolescents only and included a broad spectrum of sweet snack foods that may individually affect hypertension differently (eg, chocolate versus cakes). The fruit drinks result is similarly limited in its examination of only a young population in Iran.^43^

Our differing results across the different food groups suggest that the fructose‐containing sugars they contain may not be the primary basis of harm as noted in SSBs. This view is supported by systematic reviews and meta‐analyses of prospective cohort studies which do not show an association of fructose‐containing sugars with hypertension^10^ or related cardiometabolic diseases, such as diabetes mellitus,^101^ independent of food form. A harmful association, however, has been shown between total fructose intake and gout, independent of food form.^102^ Even so, a recent comprehensive review by Caliceti et al found conflicting evidence with regard to the pathogenesis of cardiometabolic diseases from fructose‐derived uric acid.^103^ Moreover, systematic reviews and meta‐analyses of controlled trials have failed to show a harmful effect of fructose in isocaloric substitution with other carbohydrates on hypertension^9^ or related cardiometabolic outcomes.^104^, ^105^, ^106^, ^107^, ^108^ Harmful effects have only been consistently observed in hypercaloric comparisons in which fructose supplements diets with excess calories at very high doses (>25% energy) in predominantly liquid form compared with the same diets without the excess calories,^9^, ^104^, ^105^, ^106^, ^107^, ^108^, ^109^ a condition which may be more analogous to the intake of SSBs.

## Strengths and Limitations

The strengths of our systematic review and meta‐analysis are that we identified all available prospective cohorts through a systematic search strategy, performed quantitative syntheses using 3 different types of analysis (pair‐wise highest versus lowest analysis, linear and nonlinear dose‐response analysis) and assessed the quality and strength of the evidence by using the GRADE assessment. We had a large sample size, long duration of follow‐up, and adjustment for many dietary and lifestyle factors in the included studies. Another strength is that ours is the first study that comprehensively compares all the major available food sources of fructose‐containing sugars and their association with hypertension in prospective cohort studies. Additionally, our dose‐response analyses show that the risk of incident hypertension associated with SSBs crosses the clinically important harm threshold of RR>1.10 above an intake of 1 serving/day.

Our systematic review and meta‐analysis has several limitations. First, given that the studies are observational in nature, there is the possibility for residual measured and unmeasured confounding, a reason that GRADE starts observational studies at “low” quality. Second, there was evidence of indirectness in some of the relationships with limited generalizability of our findings to other populations and geographical locations. Third, sensitivity and subgroup analyses were unable to explain the heterogeneity found for SSBs and fruit. Fourth, fruit drinks, sweet snacks, yogurt, and dairy desserts were limited by serious imprecision in the pooled risk estimates given that the 95% CIs were wide and could not rule out clinically important harm or protection. Fifth, we observed evidence of publication bias for our findings for SSBs and fruit by visual inspection of funnel plot and by formal testing. Finally, there were a limited number of cohort comparisons for several food sources of sugars with unbalanced representation of different food sources. Although SSBs are the most important source of fructose‐containing sugars by contributing 13% of total sugar intake in the Canadian diet—doubled for Americans—grains and grain products as well as sweets and desserts, 2 of the other top 10 most important food source of sugars,^17^, ^18^ were not represented. Other fruit and fruit products, such as jams, purees, and dried fruit, and dairy products, such as flavored milks, were also not represented.

Weighing these strengths and limitations using GRADE, the evidence was generally weak. We assessed our certainly in the evidence for the food sources to be “very low” for fruit drinks, sweet snacks, and dairy desserts to “low” for SSBs, 100% fruit juice, fruit, and yogurt owing to combinations of downgrades for inconsistency, indirectness, imprecision, and publication bias and upgrades for dose‐response gradient for SSBs, fruit, yogurt, and 100% fruit juice.

### Implications

Dietary guidelines have shifted from a focus on nutrient‐based recommendations to a focus on food‐ and dietary pattern–based recommendations.^110^, ^111^ The main rationale for this paradigm shift has been the recognition that a focus on nutrients misses important interactions with other nutrients and the food matrix in which the nutrients are contained and subsequently consumed.^110^ Our findings on food sources of, rather than solely, sugars support this view. The harmful association between SSBs and incident hypertension supports recommendations to limit SSBs, the most important source of sugars in the United States and Canada.^17^, ^18^ The evidence for this relationship, however, cannot necessarily be applied to other important food sources of sugars. Our findings on fruit, yogurt, dairy desserts, 100% fruit juice, fruit drinks, and sweet snacks suggest that in the context of a balanced, weight‐maintaining diet, there may not be any reason to limit these foods for the prevention of hypertension, simply owing to their sugar content. On the contrary, the recommendation to increase the intake of fruit and yogurt may contribute to better diet quality and protect against the development of hypertension, especially when included as part of a DASH (Dietary Approaches to Stop Hypertension) dietary pattern,^112^, ^113^ in which fruit (which includes 100% fruit juice) and low‐fat yogurt are important components.^114^ Our results suggest that 100% fruit juice, in moderation, might provide some of the protective nutrients from fruit which underscores the importance of examining the whole dose‐response relationship for ranges and thresholds for harmful and protective associations. On the other hand, findings for dairy desserts, with the limited research available, may not directly translate to diet recommendations. Given that people are currently not meeting their recommended intakes of fruit and vegetables^115^, ^116^ or dairy,^111^, ^116^ there is an opportunity for people in North America to increase their intake of fruit and yogurt, especially at the expense of SSBs.

An issue identified in our analysis is that a highest versus lowest analysis used routinely by prospective cohort studies and other meta‐analyses may lead to misleading results. This is because of the lack of consideration for the dose‐response association between food sources of fructose‐containing sugars and cardiometabolic disease. We showed differing results between highest versus lowest and dose‐response relationship for 100% fruit juice and dairy desserts in our analysis. The highest versus lowest analysis ignored dose‐range differences between different study populations whereas the dose‐response analysis revealed the authentic relationship with incident hypertension seen with increasing intake. Investigators of prospective cohort studies studying important food sources should consider modeling dose‐response associations with disease with a nonlinearity assessment. This will allow the identification of specific dose ranges or cutoffs for protection and harm that would have important implications for dietary guidelines and public policy. Failing to do so will only perpetuate the misinterpretation of the results and, consequently, inaccurate conclusions regarding relationships between sugar‐containing foods and important health outcomes such as hypertension.

## Conclusions

Our systematic review and meta‐analysis of the available prospective cohort studies of the relation of important food sources of fructose‐containing sugars and incident hypertension showed that the harmful association of SSBs with incident hypertension does not hold for other important food sources of fructose‐containing sugars with protective associations even noted for yogurt, fruit, and 100% fruit juice in moderate doses. These findings suggest that caution is warranted in using the evidence from SSBs as a proxy for other food sources of sugars and support the ongoing transition from nutrient‐focused recommendations to specific food‐ and dietary pattern–based recommendations insofar as they relate to sugars and hypertension. Our confidence in the estimates is weak, and additional prospective studies are needed to improve our estimates and better understand the dose‐response relationship between important food sources of fructose‐containing sugars and hypertension. There is a need for “high” quality, randomized controlled trials that give the best protection against bias and more research on other important food sources of fructose‐containing sugars, such as grain and grain products and sweets and desserts, other fruit and fruit products, and dairy and dairy products. To better understand the interactions with the whole diet, useful avenues of investigation would include research on dietary patterns and the extent to which food sources of fructose‐containing sugars in those dietary patterns contribute to the associations with hypertension.

## Sources of Funding

This work was funded by the Canadian Institutes of Health Research (funding reference number, 129920). The Diet, Digestive tract, and Disease (3‐D) Centre, funded through the Canada Foundation for Innovation (CFI) and the Ministry of Research and Innovation's Ontario Research Fund (ORF), provided the infrastructure for the conduct of this project. Khan is funded by a Toronto 3D Knowledge Synthesis and Clinical Trials foundation Postdoctoral Fellowship Award. J.L. Sievenpiper was funded by a PSI Graham Farquharson Knowledge Translation Fellowship, Canadian Diabetes Association (CDA) Clinician Scientist award, CIHR INMD/CNS New Investigator Partnership Prize, and Banting & Best Diabetes Centre Sun Life Financial New Investigator Award. None of the sponsors had a role in any aspect of the present study, including design and conduct of the study; collection, management, analysis, and interpretation of the data; and preparation, review, approval of the manuscript or decision to publish.

## Disclosures

T.A. Khan has received research support from the Canadian Institutes of Health Research (CIHR) and an unrestricted travel donation from Bee Maid Honey Ltd. He has been an invited speaker at the Calorie Control Council Annual meeting, for which he has received an honorarium. R.J. de Souza has served as an external resource person to the World Health Organization's Nutrition Guidelines Advisory Group on trans fats, saturated fats, and polyunsaturated fats. The WHO paid for his travel and accommodation to attend meetings from 2012‐2017 to present and discuss this work. He has also done contract research for the Canadian Institutes of Health Research's Institute of Nutrition, Metabolism, and Diabetes, Health Canada, and the World Health Organization for which he received remuneration. He has received speaker's fees from the University of Toronto, and McMaster Children's Hospital. He has held grants from the Canadian Foundation for Dietetic Research, Population Health Research Institute, and Hamilton Health Sciences Corporation as a principal investigator, and is a co‐investigator on several funded team grants from Canadian Institutes of Health Research. He serves as an independent director of the Helderleigh Foundation (Canada). T.M.S. Wolever and his wife are part owners and employees of INQUIS Clinical Research, Ltd. C.W.C. Kendall has received research support from the Advanced Food Materials Network, Agriculture and Agri‐Foods Canada (AAFC), Almond Board of California, Barilla, CIHR, Canola Council of Canada, International Nut and Dried Fruit Council, International Tree Nut Council Research and Education Foundation, Loblaw Brands Ltd, National Dried Fruit Trade Association, Pulse Canada, and Unilever. He has received in‐kind research support from the Almond Board of California, American Peanut Council, California Walnut Commission, Barilla, Kellogg Canada, Loblaw Companies, Quaker (Pepsico), Primo, Unico, Unilever, and WhiteWave Foods. He has received travel support/honoraria from the American Peanut Council, International Nut and Dried Fruit Council, International Pasta Organization, Oldways Preservation Trust, and Peanut Institute. He has served on the scientific advisory board for the International Tree Nut Council, International Pasta Organization, McCormick Science Institute, and Oldways Preservation Trust. He is a member of the International Carbohydrate Quality Consortium (ICQC), executive board member of the Diabetes and Nutrition Study Group (DNSG) of the European Association for the Study of Diabetes (EASD), is on the Clinical Practice Guidelines Expert Committee for Nutrition Therapy of the EASD, and is a director of the Toronto 3D Knowledge Synthesis and Clinical Trials foundation. J.L. Sievenpiper has received research support from the Canadian Foundation for Innovation, Ontario Research Fund, Province of Ontario Ministry of Research and Innovation and Science, CIHR, Diabetes Canada, PSI Foundation, Banting and Best Diabetes Centre (BBDC), American Society for Nutrition (ASN), INC International Nut and Dried Fruit Council Foundation, National Dried Fruit Trade Association, The Tate and Lyle Nutritional Research Fund at the University of Toronto, The Glycemic Control and Cardiovascular Disease in Type 2 Diabetes Fund at the University of Toronto (a fund established by the Alberta Pulse Growers), and the Nutrition Trialists Fund at the University of Toronto (a fund established by an inaugural donation from the Calorie Control Council). He has received in‐kind food donations to support a randomized controlled trial from the Almond Board of California, California Walnut Commission, American Peanut Council, Barilla, Unilever, Unico/Primo, Loblaw Companies, Quaker, Kellogg Canada, and WhiteWave Foods. He has received travel support, speaker fees, and/or honoraria from Diabetes Canada, Mott's LLP, Dairy Farmers of Canada, FoodMinds LLC, International Sweeteners Association, Nestlé, Pulse Canada, Canadian Society for Endocrinology and Metabolism (CSEM), GI Foundation, Abbott, Biofortis, ASN, Northern Ontario School of Medicine, INC Nutrition Research & Education Foundation, European Food Safety Authority (EFSA), Comité Européen des Fabricants de Sucre (CEFS), and Physicians Committee for Responsible Medicine. He has or has had ad hoc consulting arrangements with Perkins Coie LLP, Tate & Lyle, and Wirtschaftliche Vereinigung Zucker e.V. He is a member of the European Fruit Juice Association Scientific Expert Panel and Soy Nutrition Institute Scientific Advisory Committee. He is on the clinical practice guidelines expert committees of Diabetes Canada, EASD, Canadian Cardiovascular Society (CCS), and Obesity Canada. He serves or has served as an unpaid scientific advisor for the Food, Nutrition, and Safety Program (FNSP) and the Technical Committee on Carbohydrates of the International Life Science Institute (ILSI) North America. He is a member of the International Carbohydrate Quality Consortium (ICQC), executive board member of the DNSG of the EASD, and director of the Toronto 3D Knowledge Synthesis and Clinical Trials foundation. His wife is an employee of Sobeys Inc. The remaining authors have no disclosures to report. There are no patents, products in development, or marketed products to declare.

## Supporting information


**Data S1.** Sample STATA dose‐response code—SSBs intake and incident hypertension.
**Data S2.** Dose‐response raw data.
**Table S1.** Search Terms
**Table S2.** Definitions of Food Categories
**Table S3.** Confounding Variables Among the 26 Articles on Food Sources of Fructose‐Containing Sugars and Incident Hypertension
**Table S4.** Newcastle–Ottawa Scale (NOS) for Assessing the Quality of Cohort Studies
**Table S5.** Sensitivity Analysis With Systematic Removal of Each Study
**Table S6.** GRADE Assessment
**Figure S1.** Forest plot: pair‐wise meta‐analysis of SSB intake and incident hypertension.
**Figure S2.** Forest plot: pair‐wise meta‐analysis of fruit intake and incident hypertension.
**Figure S3.** Forest plot: pair‐wise meta‐analysis of yogurt intake and incident hypertension.
**Figure S4.** Forest plot: pair‐wise meta‐analysis of dairy dessert intake and incident hypertension.
**Figure S5.** Forest plot: pair‐wise meta‐analysis of 100% fruit juice intake and incident hypertension.
**Figure S6.** Forest plot: pair‐wise meta‐analysis of fruit drink intake and incident hypertension.
**Figure S7.** Forest plot: pair‐wise meta‐analysis of sweet snack intake and incident hypertension.
**Figure S8.** Dose‐response relation between sources of fructose‐containing sugars and incident hypertension with study‐specific data points.
**Figure S9.** Subgroup analyses of SSB intake and incident hypertension.
**Figure S10.** Subgroup analyses of fruit intake and incident hypertension.
**Figure S11.** Funnel plot of natural logarithm relative risk (RR) for incident hypertension comparing the highest and lowest quantiles of SSB intake.
**Figure S12.** Trim and fill funnel plot of natural logarithm relative risk (RR) for incident hypertension comparing the highest and lowest quantiles of SSB intake.
**Figure S13.** Funnel plot of natural logarithm relative risk (RR) for incident hypertension comparing the highest and lowest quantiles of fruit intake.
