# Supplementary material for: Important Food Sources of Fructose‐Containing Sugars and Incident Hypertension: A Systematic Review and Dose‐Response Meta‐Analysis of Prospective Cohort Studies
Source: J Am Heart Assoc. 2019 Dec 12;8(24):e010977. doi: 10.1161/JAHA.118.010977 (PMC6951071; doi:10.1161/JAHA.118.010977)
Supplement: Supplementary file 1 — Data S1. Sample STATA dose‐response code—SSBs intake and incident hypertension. Data S2. Dose‐response raw data. Table S1. Search Terms Table S2. Definitions of Food Categories Table S3. Confounding Variables Among the 26 Articles on Food Sources of Fructose‐Containing Sugars and Incident Hypertension Table S4. Newcastle–Ottawa Scale (NOS) for Assessing the Quality of Cohort Studies Table S5. Sensitivity Analysis With Systematic Removal of Each Study Table S6. GRADE Assessment Figure S1. Forest plot: pair‐wise meta‐analysis of SSB intake and incident hypertension. Figure S2. Forest plot: pair‐wise meta‐analysis of fruit intake and incident hypertension. Figure S3. Forest plot: pair‐wise meta‐analysis of yogurt intake and incident hypertension. Figure S4. Forest plot: pair‐wise meta‐analysis of dairy dessert intake and incident hypertension. Figure S5. Forest plot: pair‐wise meta‐analysis of 100% fruit juice intake and incident hypertension. Figure S6. Forest plot: pair‐wise meta‐analysis of fruit drink intake and incident hypertension. Figure S7. Forest plot: pair‐wise meta‐analysis of sweet snack intake and incident hypertension. Figure S8. Dose‐response relation between sources of fructose‐containing sugars and incident hypertension with study‐specific data points. Figure S9. Subgroup analyses of SSB intake and incident hypertension. Figure S10. Subgroup analyses of fruit intake and incident hypertension. Figure S11. Funnel plot of natural logarithm relative risk (RR) for incident hypertension comparing the highest and lowest quantiles of SSB intake. Figure S12. Trim and fill funnel plot of natural logarithm relative risk (RR) for incident hypertension comparing the highest and lowest quantiles of SSB intake. Figure S13. Funnel plot of natural logarithm relative risk (RR) for incident hypertension comparing the highest and lowest quantiles of fruit intake. [file JAH3-8-e010977-s001.pdf]

SUPPLEMENTAL MATERIAL

Data S1. Sample STATA dose-response code - SSBs intake and incident hypertension.

```
clear

version 15

import excel "[usedataset]", sheet("forStata") firstrow case(lower)
sort id quintile
list rr lci uci case id doseinml quintile
tab id

capture drop lnrr
capture drop lnse

gen type=2
gen lnrr=log(rr)
gen lnuci=log(uci)
gen lnlci=log(lci)
gen lnse =((lnuci-lnlci)/(2*invnorm(0.975)))
gen dose=doseinml

* scale check
sum dose, d

* linear DR
drmeta lnrr dose, data(py case) id(id) type(type) se(lnse) eform reml
lincom dose*355, eform //per 355 ml score
return list
global b0=r(estimate)
global b1: display %4.2f r(estimate)
global lci1: display %04.2f r(lb)
global uci1: display %04.2f r(ub)
global p1: display %04.3f r(p)
if $p1<0.0001 {
global p1="<0.0001"
else {
global p1="= $p1"
}
}
global captionlinear= "RR{sub:per 355 ml/per-serving} $b1 [95% CI $lci1, $uci1] {&bull} P{sub:linear} =
$p1"
drmeta_gof //goodness of fit for linear - Deviance=59.5 [lower better],  R2=0.66 [higher better]
drmeta lnrr dose, data(py case) id(id) type(type) se(lnse) eform reml
lincom dose*1, //per 1 score
global eb1=r(estimate)
display $eb1

* non-linear using splines
capture drop doses1
capture drop doses2
sum dose, d
mkspline doses = dose, nk(3) cubic displayknots
mat knots = r(knots)

*departure from linearity
drmeta lnrr doses1 doses2 , data(py case) id(id) type(type) se(lnse) reml
testparm doses2 //wald test test
global pdep0 r(p)
global pdep1: display %5.3f $pdep0
display $pdep1 //
global pnl ="P-value{sub:non-linearity}s = $pdep1"

* dose estimate non-linearity
```

```
global dl=355 //dose to show RR for 355 ml
drmeta lnrr doses1 doses2 , data(py case) id(id) type(type) se(lnse) reml
drmeta_graph, dose($dl) ref(0) matk(knots) eform list nodraw
matrix r=r(E)
global b$dl: display %4.2f r["r1","_xb"]
global lci$dl: display %04.2f r["r1","_lb"]
global uci$dl: display %04.2f r["r1","_ub"]
global rr$dl= "RR{sub:$dl g} ${b$dl} [${lci$dl}, ${uci$dl}]"
display "${rr$dl}"

global captionnl="$pnl {&bull} {it:${rr$dl}}"

tabstat  rr  dose, stat(min max)

global xtitle="xtitle(Consumption (g/day))"
global doserange= "dose(0(5)1400)"
global ytitle="ytitle(Relative Risk)"
global yscale="yscale(range(0.9 1.4)) ylabel(0.9 1 1.2 1.4, format(%5.2g))"
global yline="yline(1, lcol(red) lw(thick) lp(.))"
global title="t1title(Sugar-sweetened beverages)"

* non-linear with linear line with bubbles
drmeta lnrr doses1 doses2 , data(py case) id(id) type(type) se(lnse)  eform  reml
drmeta_graph, ref(0) matk(knots) eform addplot($eb1*d) $xtitle $doserange $ytitle $yscale $yline $title
graph addplot scatter rr dose if quintile==1,  mfcolor(gs13) mlcolor(gs10) below jitter(2) $yscale
graph addplot scatter rr dose[w=1/lnse^2] if quintile!=1, mcolor(gs15) ms(circle) below $yscale
note("$captionlinear" " " "$captionnl", size(vsmall) color(gs1) box)
graph export "ssb-spline-1a.pdf", replace

* non-linear with linear line without bubbles
drmeta lnrr doses1 doses2 , data(py case) id(id) type(type) se(lnse) reml
drmeta_graph , ref(0) matk(knots) eform addplot($eb1*d)  note("$captionlinear" " " "$captionnl",
size(vsmall) color(gs1) box)  $xtitle $doserange $ytitle $yscale $yline $title
graph export "ssb-spline-1b.pdf", replace
```

Data S2. Dose-response raw data.

Study - Cohort	Dietary Assessment	Exposure (median)	Relative Risk (95% CI)
SSBs			
Barrio-Lopez et al., 2013 – SUN [1]	Validated FFQ	0 ml/d<br>165 ml/d<br>330 ml/d	1.00 (Reference)<br>1.30 (1.10, 1.80)<br>1.60 (1.30, 2.10)
Cohen et al., 2012 – NHS [2]	Validated FFQ	12 ml/d<br>29 ml/d<br>203 ml/d<br>355 ml/d	1.00 (Reference)<br>1.02 (0.99, 1.04)<br>1.04 (1.01, 1.07)<br>1.12 (1.08, 1.17)
Cohen et al., 2012 – NHSII [2]		12 ml/d<br>29 ml/d<br>203 ml/d<br>355 ml/d	1.00 (Reference)<br>1.00 (0.96, 1.04)<br>1.07 (1.03, 1.11)<br>1.17 (1.11, 1.23)
Cohen et al., 2012 – HPFS [2]		12 ml/d<br>29 ml/d<br>203 ml/d<br>355 ml/d	1.00 (Reference)<br>0.97 (0.93, 1.02)<br>1.04 (1.00, 1.10)<br>1.06 (0.99, 1.14)
Dhingra et al., 2007 – FOC [3]	Validated FFQ	0 ml/d<br>355 ml/d<br>533 ml/d<br>710 ml/d	1.00 (Reference)<br>1.12 (0.94, 1.34)<br>1.14 (0.97, 1.32)<br>1.15 (0.92, 1.42)
Duffey et al., 2010 – CARDIA [4]	Validated SFFQ	0 ml/d<br>337 ml/d	1.00 (Reference)<br>1.06 (0.97, 1.16)
Kang et al., 2017 – KoGES [5]	Validated SFFQ	0 ml/d<br>36 ml/d<br>71 ml/d<br>143 ml/d	1.00 (Reference)<br>0.93 (0.83, 1.04)<br>1.28 (1.12, 1.48)<br>1.55 (1.18, 2.03)
Kwak et al., 2018 – KoGES [6]	Validated SFFQ	0 ml/d<br>58 ml/d<br>208 ml/d<br>875 ml/d	1.00 (Reference)<br>1.04 (0.87, 1.24)<br>1.12 (0.95, 1.33)<br>1.21 (1.02, 1.45)
Mirmiran et al. 2015 – TLGS [7]	Validated SFFQ	1 ml/d<br>9 ml/d<br>33 ml/d<br>100 ml/d	1.00 (Reference)<br>0.80 (0.27, 2.33)<br>1.35 (0.50, 3.51)<br>2.59 (1.05, 5.97)
Sayon-Orea et al., 2015 – SUN [8]	Validated SFFQ	0 ml/d<br>99 ml/d<br>198 ml/d	1.00 (Reference)<br>1.07 (0.94, 1.22)<br>1.34 (1.09, 1.65)
Weng et al., 2013 – ARIC [9]	Validated FFQ	0 ml/d<br>178 ml/d<br>355 ml/d	1.00 (Reference)<br>1.11 (1.01, 1.23)<br>1.02 (0.90, 1.16)
Winkelmayer et al., 2005 – NHS [10]	Validated FFQ	0 ml/d<br>355 ml/d<br>888 ml/d<br>1420 ml/d	1.00 (Reference)<br>1.09 (0.98, 1.22)<br>1.11 (0.95, 1.30)<br>1.44 (0.98, 2.11)
Winkelmayer et al., 2005 – NHSII [10]		0 ml/d<br>355 ml/d<br>888 ml/d<br>1420 ml/d	1.00 (Reference)<br>1.13 (1.03, 1.24)<br>1.24 (1.11, 1.38)<br>1.28 (1.01, 1.62)
Fruit			
Auerbach et al., 2017 – WHI [11]	Validated SFFQ	26 g/d<br>61 g/d	1.00 (Reference)<br>1.00 (0.99, 1.04)

		79 g/d	1.01 (0.98, 1.04)
		140 g/d	1.00 (0.97, 1.03)
		210 g/d	1.02 (0.98, 1.04)
Borgi et al., 2016 – NHS [12]	Validated FFQ	50 g/d	1.00 (Reference)
		69 g/d	0.97 (0.93, 1.01)
		88 g/d	0.95 (0.92, 0.99)
		219 g/d	0.94 (0.91, 0.98)
		350 g/d	0.96 (0.88, 1.03)
Borgi et al., 2016 – NHSII [12]		50 g/d	1.00 (Reference)
		69 g/d	1.03 (0.99, 1.07)
		88 g/d	0.97 (0.94, 1.07)
		219 g/d	0.91 (0.87, 0.95)
		350 g/d	0.91 (0.81, 1.02)
Borgi et al., 2016 – HPFS [12]		50 g/d	1.00 (Reference)
		69 g/d	0.95 (0.89, 1.00)
		88 g/d	0.92 (0.88, 0.97)
		219 g/d	0.92 (0.87, 0.97)
		350 g/d	0.88 (0.81, 0.97)
Kim et al. J Acad Nutr, 2017 – KoGES [13] (men)	Validated SFFQ	0 g/d	1.00 (Reference)
		150 g/d	0.58 (0.45, 0.75)
		300 g/d	0.44 (0.34, 0.57)
		400 g/d	0.44 (0.32, 0.60)
Kim et al. J Acad Nutr, 2017 – KoGES [13] (women)		0 g/d	1.00 (Reference)
		150 g/d	0.71 (0.54, 0.95)
		300 g/d	0.44 (0.33, 0.58)
		400 g/d	0.33 (0.24, 0.45)
Koochakpoor et al., 2018 – TLGS [14]	Validated SFFQ	0 g/d	1.00 (Reference)
		80 g/d	0.83 (0.68, 1.40)
		200 g/d	0.97 (0.58, 1.77)
		320 g/d	0.89 (0.63, 1.30)
Nunez-Cordoba et al., 2009 – SUN [15]	Validated SFFQ	160 g/d	1.00 (Reference)
		248 g/d	0.86 (0.66, 1.13)
		408 g/d	0.94 (0.70, 1.27)
		568 g/d	1.02 (0.72, 1.27)
		640 g/d	0.85 (0.59, 1.22)
Psaltopoulou et al., 2004 – EPIC [16]	Validated SFFQ	106 g/d	1.00 (Reference)
		318 g/d	0.61 (0.45, 0.83)
Steffen et al., 2005 – CARDIA [17]	Validated SFFQ	2 g/d	1.00 (Reference)
		31 g/d	0.88 (0.72, 1.06)
		61 g/d	0.83 (0.68, 1.01)
		105 g/d	0.85 (0.69, 1.04)
		131 g/d	0.75 (0.60, 0.94)
Tsubota-Utsugi et al., 2011 – Ohasama [18]	Validated FFQ	38 g/d	1.00 (Reference)
		51 g/d	0.73 (0.46, 1.09)
		82 g/d	0.78 (0.50, 1.16)
		100 g/d	0.51 (0.29, 0.81)
Wang et al., 2012 – WHS [19]	Validated FFQ	44 g/d	1.00 (Reference)
		88 g/d	0.99 (0.92, 1.06)
		153 g/d	0.98 (0.90, 1.06)
		219 g/d	0.98 (0.91, 1.06)
		263 g/d	0.95 (0.88, 1.04)
Weng et al., 2013 – ARIC [9]	Validated FFQ	89 g/d	1.00 (Reference)
		123 g/d	1.06 (0.94, 1.19)
		153 g/d	0.98 (0.87, 1.10)
		181 g/d	1.08 (0.96, 1.22)

		225 g/d	1.06 (0.93, 1.20)
Yogurt			
Alonso et al., 2009 – ARIC [20]	Validated FFQ	2 g/d<br>74 g/d<br>319 g/d	1.00 (Reference)<br>1.01 (0.89, 1.14)<br>1.11 (0.86, 1.41)
Buendia et al., 2018 – NHS [21]	Validated SFFQ	7 g/d<br>22 g/d<br>53 g/d<br>123 g/d<br>175 g/d	1.00 (Reference)<br>1.00 (0.97, 1.02)<br>0.99 (0.96, 1.01)<br>0.95 (0.92, 0.98)<br>0.87 (0.81, 0.94)
Buendia et al., 2018 – NHSII [21]		7 g/d<br>22 g/d<br>53 g/d<br>123 g/d<br>175 g/d	1.00 (Reference)<br>0.96 (0.93, 1.00)<br>0.95 (0.92, 0.98)<br>0.93 (0.90, 0.97)<br>0.89 (0.82, 0.96)
Buendia et al., 2018 – HPFS [21]		7 g/d<br>22 g/d<br>53 g/d<br>123 g/d<br>175 g/d	1.00 (Reference)<br>0.98 (0.93, 1.02)<br>0.94 (0.89, 0.99)<br>0.95 (0.89, 1.01)<br>1.01 (0.89, 1.15)
Engberink et al., 2009 – MORGEN [22]	Validated SFFQ	12 g/d<br>29 g/d<br>70 g/d<br>122 g/d	1.00 (Reference)<br>0.91 (0.74, 1.09)<br>0.86 (0.71, 1.05)<br>0.91 (0.74, 1.09)
Kim et al., Brit J Nutr, 2017 – KoGES [23]	Validated SFFQ	0 g/d<br>20 g/d<br>49 g/d<br>78 g/d	1.00 (Reference)<br>0.67 (0.58, 0.76)<br>0.71 (0.62, 0.81)<br>0.71 (0.59, 0.85)
Steffen et al., 2005 – CARDIA [24]	Validated SFFQ	4 g/d<br>11 g/d<br>18 g/d	1.00 (Reference)<br>1.00 (0.83, 1.20)<br>0.88 (0.75, 1.04)
Wang et al., 2008 – WHS [17]	Validated SFFQ	8 g/d<br>16 g/d<br>88 g/d<br>193 g/d	1.00 (Reference)<br>0.95 (0.90, 1.01)<br>0.95 (0.89, 1.01)<br>0.93 (0.81, 1.07)
Wang et al., 2015 – FHS [25]	Validated FFQ	0 g/d<br>227 g/d	1.00 (Reference)<br>0.95 (0.90, 0.99)
Dairy Desserts			
Alonso et al., 2009 – ARIC [17]	Validated FFQ	14 ml/d<br>273 ml/d<br>533 ml/d	1.00 (Reference)<br>0.91 (0.83, 0.99)<br>0.88 (0.74, 1.04)
Steffen et al., 2005 – CARDIA [20]	Validated SFFQ	4 ml/d<br>21 ml/d<br>40 ml/d<br>72 ml/d<br>93 ml/d	1.00 (Reference)<br>0.81 (0.67, 0.98)<br>0.87 (0.71, 1.05)<br>0.79 (0.65, 0.97)<br>0.74 (0.60, 0.92)
Wang et al., 2008 – WHS [22]	Validated SFFQ	8 ml/d<br>17 ml/d<br>89 ml/d<br>196 ml/d<br>250 ml/d	1.00 (Reference)<br>1.08 (1.02, 1.14)<br>1.04 (0.97, 1.10)<br>1.13 (0.98, 1.31)<br>0.90 (0.76, 1.07)
100% Fruit Juice			
Auerbach et al., 2017 – WHI [4]	Validated SFFQ	0 ml/d<br>30 ml/d<br>77 ml/d	1.00 (Reference)<br>0.98 (0.94, 1.01)<br>0.97 (0.94, 1.01)

		145 ml/d<br>231 ml/d	0.98 (0.94, 1.01)<br>1.01 (0.97, 1.04)
Duffey et al., 2010 – CARDIA [13]	Validated SFFQ	0 ml/d<br>114 ml/d	1.00 (Reference)<br>0.89 (0.82, 0.97)
Fruit Drinks			
Mirmiran et al. 2015 – TLGS [7]	Validated SFFQ	1 ml/d<br>8 ml/d<br>20 ml/d<br>67 ml/d	1.00 (Reference)<br>2.00 (0.71, 5.66)<br>1.91 (0.65, 5.60)<br>1.28 (0.04, 3.94)
Sweet Snacks			
Asghari et al., 2016 – TLGS [26]	Validated SFFQ	7 g/d<br>19 g/d<br>35 g/d<br>73 g/d	1.00 (Reference)<br>1.17 (0.45, 3.93)<br>2.49 (0.82, 7.59)<br>2.18 (0.70, 6.81)

Table S1. Search terms.

MEDLINE		EMBASE		Cochrane	
1	sugar*.mp.	1	sugar*.mp.	1	sugar*.mp.
2	exp fructose/	2	exp sugar/	2	exp fructose/
3	fructose.mp.	3	exp fructose/	3	fructose.mp.
4	HFCS.mp.	4	fructose.mp.	4	HFCS.mp.
5	exp High Fructose Corn Syrup/	5	HFCS.mp.	5	exp Nutritive Sweeteners/
6	sucrose.mp.	6	exp high fructose corn syrup/	6	sucrose.mp.
7	exp Dietary Sucrose/	7	sucrose.mp.	7	exp dietary sucrose/
8	sugar sweetened beverage*.mp.	8	exp dietary sucrose/	8	sugar sweetened beverage*.mp.
9	SSB.mp.	9	sugar sweetened beverage*.mp.	9	ssb.mp.
10	soda.mp.	10	SSB.mp.	10	soda.mp.
11	soft drink*.mp.	11	soda.mp.	11	soft drink*.mp.
12	exp Carbonated Beverages/	12	soft drink*.mp.	12	exp carbonated beverages/
13	carbonated beverages.mp.	13	exp soft drink/	13	non alcoholic beverage*.mp.
14	non alcoholic beverage*.mp.	14	exp Carbonated Beverages/	14	nonalcoholic beverage*.mp.
15	nonalcoholic beverage*.mp.	15	carbonated beverages.mp.	15	exp energy drinks/
16	exp Energy Drinks/	16	non alcoholic beverage*.mp.	16	energy drink*.mp.
17	energy drink*.mp.	17	nonalcoholic beverage*.mp.	17	smoothie*.mp.
18	smoothie*.mp.	18	exp energy drink/	18	((fruit or vegetable) and juice*).mp.
19	exp "Fruit and Vegetable Juices"/	19	energy drink*.mp.	19	fruit.mp.
20	fruit.mp.	20	smoothie*.mp.	20	exp fruit/
21	exp Fruit/	21	exp "fruit and vegetable juice"/	21	exp honey/
22	exp Honey/	22	fruit.mp.	22	y*g*rt.mp.
23	y*g*rt.mp.	23	exp fruit/	23	exp yogurt/
24	exp Yogurt/	24	exp honey/	24	ice cream*.mp.
25	ice cream*.mp.	25	y*g*rt.mp.	25	icecream*.mp.
26	icecream*.mp.	26	exp yoghurt/	26	exp ice cream/
27	exp Ice Cream/	27	exp ice cream/	27	cereal*.mp.
28	cereal*.mp.	28	ice cream*.mp.	28	dessert*.mp.
29	exp edible grain/	29	icecream*.mp.	29	sweets.mp.
30	dessert*.mp.	30	cereal*.mp.	30	confection*.mp.
31	sweets.mp.	31	dessert*.mp.	31	pastries.mp.
32	confection*.mp.	32	sweets.mp.	32	biscuit*.mp.
33	pastries.mp.	33	confection*.mp.	33	cookie*.mp.
34	biscuit*.mp.	34	exp bakery product/	34	cake*.mp.
35	cookie*.mp.	35	pastries.mp.	35	candy.mp.
36	cake*.mp.	36	biscuit*.mp.	36	candies.mp.
37	candy.mp.	37	cookie*.mp.	37	exp candy/

Table S1. Search terms (Continued)

	MEDLINE		EMBASE		Cochrane
38	candies.mp.	38	cake*.mp.	38	(chocolate adj2 milk).mp.
39	exp Candy/	39	candy.mp.	39	chocolate.mp
40	(chocolate adj2 milk).mp.	40	candies.mp.	40	exp cacao/
41	exp chocolate/	41	(chocolate adj2 milk).mp.	41	cacao.mp
42	chocolate.mp	42	exp chocolate/	42	or/1-41
43	exp cacao/	43	chocolate.mp	43	cohort.mp.
44	cacao.mp	44	exp cacao/	44	exp Prospective Studies/
45	or/1-44	45	cacao.mp	45	(prospective adj2 (cohort or study)).mp.
46	cohort.mp.	46	or/1-45	46	exp follow-up studies/
47	exp prospective study/	47	cohort.mp.	47	exp multivariate analysis/
48	(prospective adj2 (cohort or study)).mp.	48	exp prospective study/	48	exp proportional hazards models/
49	exp Follow-Up Studies/	49	(prospective adj2 (cohort or study)).mp.	49	follow up study.mp.
50	exp Multivariate Analysis/	50	exp multivariate analysis/	50	(longitudinal adj2 study).mp.
51	exp Proportional Hazards Models/	51	exp proportional hazards model/	51	or/43-50
52	follow up study.mp.	52	follow up study.mp.	52	hypertensive*.mp.
53	(longitudinal adj2 study).mp.	53	(longitudinal adj2 study).mp.	53	exp Hypertension/
54	or/46-53	54	or/47-53	54	hypertension*.mp.
55	hypertensive*.mp.	55	hypertensive*.mp.	55	HTN.mp.
56	exp Hypertension/	56	exp Hypertension/	56	blood pressure.mp.
57	hypertension*.mp.	57	hypertension*.mp.	57	exp Blood Pressure/
58	HTN.mp.	58	HTN.mp.	58	systolic blood pressure.mp.
59	blood pressure.mp.	59	blood pressure.mp.	59	SBP.mp.
60	exp Blood Pressure/	60	exp Blood Pressure/	60	diastolic blood pressure.mp.
61	systolic blood pressure.mp.	61	systolic blood pressure.mp.	61	DBP.mp.
62	SBP.mp.	62	SBP.mp.	62	or/52-61
63	diastolic blood pressure.mp.	63	diastolic blood pressure.mp.	63	and/42,51,62
64	DBP.mp.	64	DBP.mp.		
65	or/55-64	65	or/55-64		
66	and/45,54,65	66	and/46,54,65		

Database	Total
MEDLINE: December week 2 2018	1,063
EMBASE: December week 2 2018	2,428
Cochrane: December week 2 2018	173
Manual search	5
**Total**	**3,669**

The original search was conducted November week 1 2016. The search was updated twice, to December week 2 2018.

Table S2. Definitions of food categories.

Sugar-sweetened beverages (SSBs)	
Barrio-Lopez et al., 2013 – SUN [1]	Sugar-sweetened carbonated colas; fruit-flavoured carbonated sugar soft drinks
Cohen et al., 2012 – NHS, NHSII, HPFS [2]	Sugar-sweetened cola; sugar-sweetened caffeine-free cola; sugar-sweetened non-cola; and fruit punch or other sugar-sweetened fruit drink
Dhingra et al., 2007 – FOC [3]	Soft drinks (Coke, Pepsi, Sprite, or other carbonated soft drinks) – caffeinated or non-caffeinated
Duffey et al., 2010 – CARDIA [4]	Sugar-sweetened soda; fruit drinks
Kang et al., 2017 – KoGES [5]	Soft drinks (carbonated beverages, e.g., Cola and Sprite)
Kwak et al., 2018 – KoGES [6]	Soft drinks (coke or sprite) and other sweetened drinks (sweetened rice drink and sweetened citrus tea)
Mirmiran et al., 2015 – TLGS [7]	Sugar-sweetened carbonated soft drinks
Sayon-Orea et al., 2015 – SUN [8]	Sugar-sweetened carbonated colas; fruit-flavored carbonated sugar soft drinks
Weng et al., 2013 – ARIC [9]	Not specified
Winkelmayer et al., 2005 – NHS, NHSII [10]	Regular cola (Coke, Pepsi, or other cola beverages with sugar)
Fruit	
Borgi et al., 2016 – NHS, NHSII, HPFS [11]	Whole fruits: raisins/grapes; fresh apples/pears; bananas; strawberries; blueberries; prunes; avocado; cantaloupe; oranges; peaches/apricots/plums
Kim et al., J Acad Nutr Diet, 2017 – KoGES [12]	Tangerines, oranges, persimmon or dried persimmon, watermelon, strawberry, grape, pear, oriental melon/melon, peach or prune, apple, banana, and tomato
Auerbach et al., 2017 – WHI [13]	Not specified
Koochakpoor et al., 2018 – TLGS [14]	
Nunez-Cordoba et al., 2009 – SUN [15]	
Psaltopoulou et al., 2004 – EPIC [16]	
Steffen et al., 2005 – CARDIA [17]	
Tsubota-Utsugi et al., 2011 – Ohasama [18]	
Wang et al., 2012 – WHS [19]	
Weng et al., 2013 – ARIC [9]	
Yogurt	
Wang et al., 2008 – WHS [20]	Low-fat yogurt
Buendia et al., 2018 – NHS, NHSII, HPFS [21]	Yogurt (all types)
Alonso et al., 2009 – ARIC [22]	Not specified
Engberink et al., 2009 – MORGEN [23]	
Kim et al., Brit J Nutr, 2017 – KoGES [24]	
Steffen et al., 2005 – CARDIA [17]	
Wang et al., 2015 – FHS [25]	
Dairy desserts	
Steffen et al., 2005 – CARDIA [17]	Dairy desserts (not specified)
Wang et al., 2008 – WHS [20]	Low-fat sherbet
Alonso et al., 2009 – ARIC [22]	Ice cream
100% Fruit juice	
Duffey et al., 2010 – CARDIA [4]	Fruit juice (non-sweetened)
Auerbach et al., 2017 – WHI [13]	100% fruit juice
Fruit drinks	
Mirmiran et al., 2015 – TLGS [7]	Fruit juice – sugar-sweetened drinks and non-sweetened
Sweet snacks	
Asghari et al., 2016 – TLGS [26]	Candies, chocolates, cookies, cakes, biscuits, confectionery, caramels, and traditional Iranian confectioneries, such as gaz, sohan, noghl, halva, Yazdi cakes

Table S3. Confounding variables among the 26 articles on food sources of fructose-containing sugars and incident hypertension.

Cohort Study	Alonso et al., 2009 – ARIC [22]	Asghari et al., 2016 – TLGS [26]	Auerbach et al., 2017 – WHI [13]	Buendia et al., 2018 – HPFS, NHS, NHSII [21]	Barrio-Lopez et al., 2013 – SUN [1]	Borgi et al., 2016 – HPFS [11]	Borgi et al., 2016 – NHSII [11]	Borgi et al., 2016 - NHS [11]	Cohen et al., 2012 – HPFS [2]	Cohen et al., 2012 – NHSII [2]	Cohen et al., 2012 – NHS [2]	Dhingra et al., 2007 - FOC [3]	Duffey et al., 2010 – CARDIA [4]	Engberink et al., 2009 – MORGEN [23]	Kang et al., 2017 – KoGES [5]	Kim et al., Brit J Nutr, 2017 – KoGES [24]	Kim et al., J Acad Nutr Diet, 2017 – KoGES [12]	Koochakpoor et al., 2018 – TLGS [14]	Kwak et al., 2018 – KoGES [6]	Mirmiran et. al., 2015 – TLGS [7]	Nunez-Cordoba et al., 2009 – SUN [15]	Psaltopoulou et al., 2004 – EPIC [16]	Sayon-Orea et al., 2015 – SUN [8]	Steffen et al., 2005 – CARDIA [17]	Tsubota-Utsugi et al., 2011 - Ohasama [18]	Wang et al., 2008 – WHS [20]	Wang et al., 2012 – WHS [19]	Wang et al., 2015 – FHS [25]	Weng et al., 2013 – ARIC [9]	Winkelmayer et al., 2005 – NHS [10]	Winkelmayer et al., 2005 – NHSII [10]
**Number of variables in fully adjusted model**	14	7	14	11	12	15	17	16	21	22	22	11	12	13	13	12	16	8	15	11	12	10	22	10	12	16	17	10	9	8	9
**Number of multivariable models presented**	1	2	1	2	1	1	1	1	2	2	2	1	1	3	2	2	3	1	3	3	1	1	2	1	2	2	4	3	3	1	1
**Timing of measurement of confounding variables**	BL	BL	BL	Every 2y	BL	BL*	BL*	BL*	BL*	BL*	BL*	BL	BL	BL	BL†	BL†	BL†	BL	Every 2y	BL	BL	BL	BL	BL	BL	BL	BL	Every exam ‡	BL	Every 2y	Every 2y
**Pre-specified primary confounding variable**																															
Age	✓	✓	✓	✓	✓	✓	✓	✓	✓	✓	✓	✓	✓	✓	✓	✓	✓	✓	✓	✓	✓	✓	✓	✓	✓	✓	✓	✓	✓	✓	✓
**Pre-specified secondary confounding variables**																															
Smoking	✓		✓	✓	✓	✓	✓	✓	✓	✓	✓	✓	✓	✓	✓	✓	✓	✓	✓		✓		✓	✓	✓	✓	✓	✓	✓	✓	✓
Markers of overweight/obesity (body mass index, weight, waist circumference, waist to hip ratio)	✓§	✓	✓	✓	✓	✓	✓	✓	✓	✓	✓		✓	✓	✓	✓	✓	✓		✓	✓	✓§	✓		✓	✓	✓	✓		✓	✓
Energy intake	✓	✓	✓	✓	✓	✓	✓	✓	✓	✓	✓	✓	✓	✓	✓	✓	✓	✓	✓	✓	✓	✓	✓	✓		✓	✓	✓	✓		
Physical activity	✓	✓	✓	✓	✓	✓	✓	✓	✓	✓	✓	✓	✓		✓	✓	✓	✓	✓	✓	✓		✓	✓	✓	✓	✓	✓	✓	✓	✓
Sex	✓	✓	F‖	F‖/ M#	✓	M#	F‖	F‖	M#	F‖	F‖	✓	✓	✓	✓	✓	✓	✓	✓	✓	✓	✓	✓	✓	✓	F‖	F‖	✓	✓	F‖	F‖
Diabetes			✓												✓				✓						✓	✓	✓				
Alcohol consumption	✓				✓	✓	✓	✓	✓	✓	✓		✓	✓	✓	✓	✓		✓		✓		✓	✓	✓	✓	✓			✓	✓
Sodium intake	✓		✓																✓		✓		✓		✓				✓		
**Other confounding variables**																															
Family history of HTN				✓		✓	✓	✓	✓	✓	✓										✓		✓							✓	✓
Attempting to lose weight									✓	✓	✓																				
Baseline blood pressure												✓													✓						
Baseline soft drink intake																															
Change in weight						✓	✓	✓	✓	✓	✓																				
Diet:																															
DASH style diet									✓	✓	✓																				

Cohort Study	Alonso et al., 2009 – ARIC [22]	Asghari et al., 2016 – TLGS [26]	Auerbach et al., 2017 – WHI [13]	Buendia et al., 2018 – HPFS, NHS, NHSII [21]	Barrio-Lopez et al., 2013 – SUN [1]	Borgi et al., 2016 – HPFS [11]	Borgi et al., 2016 – NHSII [11]	Borgi et al., 2016 - NHS [11]	Cohen et al., 2012 – HPFS [2]	Cohen et al., 2012 – NHSII [2]	Cohen et al., 2012 – NHS [2]	Dhingra et al., 2007 - FOC [3]	Duffey et al., 2010 – CARDIA [4]	Engberink et al., 2009 – MORGEN [23]	Kang et al., 2017 – KoGES [5]	Kim et al., Brit J Nutr, 2017 – KoGES [24]	Kim et al., J Acad Nutr Diet, 2017 – KoGES [12]	Koochakpoor et al., 2018 – TLGS [14]	Kwak et al., 2018 – KoGES [6]	Mirmiran et. al., 2015 – TLGS [7]	Nunez-Cordoba et al., 2009 – SUN [15]	Psaltopoulou et al., 2004 – EPIC [16]	Sayon-Orea et al., 2015 – SUN [8]	Steffen et al., 2005 – CARDIA [17]	Tsubota-Utsugi et al., 2011 - Ohasama [18]	Wang et al., 2008 – WHS [20]	Wang et al., 2012 – WHS [19]	Wang et al., 2015 – FHS [25]	Weng e al., 2013 – ARIC [9]	Winkelmayer et al., 2005 – NHS [10]	Winkelmayer et al., 2005 – NHSII [10]
Modified Dietary Guidelines Adherence Index (DGAI) score																												✓			
Mediterranean diet adherence					✓																										
Healthy Eating Index (HEI) score			✓																												
Energy from other beverages:																															
ASBs						✓	✓	✓	✓	✓	✓																			✓	✓
Caffeinated tea, coffee														✓						✓										✓	✓
Caffeinated coffee																												✓			
Fruit juice													✓**																		
Low fat milk													✓																		
SSBs						✓	✓	✓					✓**																		
Whole fat milk													✓																		
Bread														✓																	
Calcium									✓	✓	✓					✓															
Carbohydrates									✓	✓	✓																				
Glycemic index												✓																			
Total fructose									✓	✓	✓																				
Cereals																							✓								
Fast food					✓																										
Fat															✓										✓						
Saturated fat												✓																			
Trans fat									✓	✓	✓	✓																			
Fiber		✓							✓	✓	✓	✓			✓	✓				✓											
French fries					✓																										
Fruit	✓			✓										✓						✓			✓			✓					
Legumes																	✓						✓								
Low fat dairy																					✓		✓				✓				
Whole fat dairy																			✓				✓								
Total Dairy																	✓		✓												

Cohort Study	Alonso et al., 2009 – ARIC [22]	Asghari et al., 2016 – TLGS [26]	Auerbach et al., 2017 – WHI [13]	Buendia et al., 2018 – HPFS, NHS, NHSII [21]	Barrio-Lopez et al., 2013 – SUN [1]	Borgi et al., 2016 – HPFS [11]	Borgi et al., 2016 – NHSII [11]	Borgi et al., 2016 - NHS [11]	Cohen et al., 2012 – HPFS [2]	Cohen et al., 2012 – NHSII [2]	Cohen et al., 2012 – NHS [2]	Dhingra et al., 2007 - FOC [3]	Duffey et al., 2010 – CARDIA [4]	Engberink et al., 2009 – MORGEN [23]	Kang et al., 2017 – KoGES [5]	Kim et al., Brit J Nutr, 2017 – KoGES [24]	Kim et al., J Acad Nutr Diet, 2017 – KoGES [12]	Koochakpoor et al., 2018 – TLGS [14]	Kwak et al., 2018 – KoGES [6]	Mirmiran et. al., 2015 – TLGS [7]	Nunez-Cordoba et al., 2009 – SUN [15]	Psaltopoulou et al., 2004 – EPIC [16]	Sayon-Orea et al., 2015 – SUN [8]	Steffen et al., 2005 – CARDIA [17]	Tsubota-Utsugi et al., 2011 - Ohasama [18]	Wang et al., 2008 – WHS [20]	Wang et al., 2012 – WHS [19]	Wang et al., 2015 – FHS [25]	Weng e al., 2013 – ARIC [9]	Winkelmayer et al., 2005 – NHS [10]	Winkelmayer et al., 2005 – NHSII [10]
Magnesium									✓	✓	✓	✓																			
Meat/meat products/animal flesh					✓	✓	✓	✓						✓			✓														
Fish														✓							✓		✓								
Red meat					✓															✓			✓			✓	✓				
Nuts																											✓				
Olive oil																							✓								
Potassium	✓																✓		✓				✓								
Protein intake				✓																											
Vegetables	✓			✓		✓	✓	✓						✓						✓			✓			✓	✓				
Vitamin D									✓	✓	✓																				
Whole grains						✓	✓	✓									✓		✓		✓					✓	✓				
Vitamin use																								✓		✓	✓				
Medical history																															
CVD															✓	✓	✓		✓						✓						
Family History of Diabetes		✓																		✓											
Hypercholesterolemia																									✓	✓	✓				
Menopausal status			✓				✓	✓																		✓	✓				
Non-narcotic analgesics use						✓	✓	✓	✓	✓	✓																				
Oral contraceptive use							✓			✓	✓																				✓
Post-menopausal hormone use			✓																								✓				
Socio-economic status														✓		✓		✓													
Education			✓												✓		✓		✓			✓	✓	✓					✓		
Income															✓		✓		✓												
Ethno-cultural/geographical factors																															
Ethnicity			✓	✓		✓	✓	✓	✓	✓	✓		✓											✓		✓	✓		✓		
Exam center	✓												✓											✓					✓		
Study visit	✓																														
Residence (urban vs. rural)																✓	✓					✓									
Others																															
(Alcohol)[2]																							✓								

Cohort Study	Alonso et al., 2009 – ARIC [22]	Asghari et al., 2016 – TLGS [26]	Auerbach et al., 2017 – WHI [13]	Buendia et al., 2018 – HPFS, NHS, NHSII [21]	Barrio-Lopez et al., 2013 – SUN [1]	Borgi et al., 2016 – HPFS [11]	Borgi et al., 2016 – NHSII [11]	Borgi et al., 2016 - NHS [11]	Cohen et al., 2012 – HPFS [2]	Cohen et al., 2012 – NHSII [2]	Cohen et al., 2012 – NHS [2]	Dhingra et al., 2007 - FOC [3]	Duffey et al., 2010 – CARDIA [4]	Engberink et al., 2009 – MORGEN [23]	Kang et al., 2017 – KoGES [5]	Kim et al., Brit J Nutr, 2017 – KoGES [24]	Kim et al., J Acad Nutr Diet, 2017 – KoGES [12]	Koochakpoor et al., 2018 – TLGS [14]	Kwak et al., 2018 – KoGES [6]	Mirmiran et. al., 2015 – TLGS [7]	Nunez-Cordoba et al., 2009 – SUN [15]	Psaltopoulou et al., 2004 – EPIC [16]	Sayon-Orea et al., 2015 – SUN [8]	Steffen et al., 2005 – CARDIA [17]	Tsubota-Utsugi et al., 2011 - Ohasama [18]	Wang et al., 2008 – WHS [20]	Wang et al., 2012 – WHS [19]	Wang et al., 2015 – FHS [25]	Weng e al., 2013 – ARIC [9]	Winkelmayer et al., 2005 – NHS [10]	Winkelmayer et al., 2005 – NHSII [10]
(BMI)^2									✓	✓	✓																				
Interactions btwn: (age and residence), (age and sex), (sex and residence)																						✓									
Interactions between: (follow-up time and physical activity), (follow-up time and age)																												✓			
Randomized treatment			✓																							✓	✓				
SNP for cyclin D2 polymorphism																		✓													

BL = Confounders measured only at baseline year
* Baseline for all confounders except for [change in weight], which was per food frequency questionnaire cycle
† Baseline for all confounders except for dietary confounders, which was assessed at baseline and follow-up
‡ Exams were (1991-1995), (1995-1998), (1998-2001), (2005-2008)
§ Both BMI and waist-to-hip ratio were controlled for
|| Indicates the study includes only female subjects
# Indicates the study includes only male subjects
** Fruit juice analysis controlled for SSB intake, whereas SSB analysis controlled for fruit juice intake
✓ Means variable adjusted for in the most adjusted model.

Table S4. Newcastle-Ottawa Scale (NOS) for Assessing the Quality of Cohort Studies.

Study	Selection*	Outcome †	Comparability ‡	Total §
Alonso et al., 2009 [22]	4	3	2	9
Asghari et al., 2016 [26]	4	3	1	8
Auberbach et al., 2017 [13]	3	1	2	6
Barrio-Lopez et al., 2013 [1]	3	3	2	7
Borgi et al., 2016 [11]	3	2	2	6
Buenda et al., 2018 [21]	2	2	1	5
Cohen et al., 2012 [2]	3	2	2	6
Dhingra et al., 2007 [3]	4	2	1	6
Duffey et al., 2010 [4]	4	1	2	7
Engberink et al., 2009 [23]	3	2	1	6
Kang et al., 2017 [5]	4	2	2	8
Kim et al., Br J Nutr, 2017 [24]	4	2	2	8
Kim et al., J Acad Nutr Diet, 2017 [12]	4	2	2	8
Koochakpoor et al., 2018 [14]	4	3	1	8
Kwak et al., 2018 [6]	4	2	2	8
Mirmiran et al., 2015 [7]	4	3	1	8
Nun˜ez-Cordoba et al., 2009 [15]	3	3	2	7
Psaltopoulou et al., 2004 [16]	3	2	1	6
Sayon-Orea et al., 2015 [8]	3	2	2	6
Steffen et al., 2005 [17]	4	2	1	7
Tsubota-Utsugi et al., 2011 [18]	4	2	2	7
Wang et al., 2008 [20]	3	2	2	6
Wang et al., 2012 [19]	3	1	2	5
Wang et al., 2015 [9]	3	2	1	6
Weng et al., 2013 [9]	4	3	1	8
Winkelmayer et al., 2005 [10]	3	3	1	6

* Maximum 4 points awarded for cohort representativeness, selection of non-exposed cohort, exposure assessment and demonstration outcome not present at baseline.

† Maximum 3 points awarded for follow-up length, adequacy of follow-up and outcome assessment.

‡ Maximum 2 points awarding for controlling for the pre-specified primary confounding variable (age) and ≥ 6 of the secondary confounding variables (sex, any marker of adiposity, smoking, energy intake, physical activity, diabetes/dysglycemia, alcohol intake, sodium intake).

§ A maximum of 9 points could be awarded.

Table S5. Sensitivity analysis with systematic removal of each study.

Removal of:	Participants N	Cases N	Risk Ratio for Incident Hypertension RR	95% CI	p-value	Heterogeneity I²	p-value
**SSBs**							
All included:	427,630	120,553	1.17	[1.11, 1.23]	<0.00001	66%	0.0004
Barrio-Lopez, Brit J Nutr, 2013 - SUN	8157	1464	1.14	[1.09, 1.20]	<0.00001	58%	0.006
Cohen, J Gen Intern Med, 2012 - HPFS	37360	13439	1.19	[1.12, 1.26]	<0.00001	66%	0.0006
Cohen, J Gen Intern Med, 2012 - NHS	88540	42022	1.19	[1.11, 1.28]	<0.00001	66%	0.0002
Cohen, J Gen Intern Med, 2012 - NHSII	97991	21873	1.18	[1.10, 1.25]	<0.00001	67%	0.0005
Dhingra, Circulation, 2007 - FOC	2803	1377	1.17	[1.11, 1.24]	<0.00001	69%	0.0002
Duffrey, Am J Clin Nutr, 2010 - CARDIA	2639	609	1.19	[1.12, 1.27]	<0.00001	63%	0.002
Kang, Brit J Nutr, 2017 - KoGES	4591	1309	1.15	[1.09, 1.21]	<0.00001	64%	0.001
Kwak, Eur J Nutr, 2018 - KoGES	5775	1175	1.17	[1.10, 1.24]	<0.00001	69%	0.0003
Mirmiran, Nutr Metab, 2015 - TLGS	424	47	1.16	[1.10, 1.22]	<0.00001	65%	0.0008
Sayon-Orea, Clin Nutr, 2015 - SUN	13843	1308	1.16	[1.10, 1.22]	<0.00001	67%	0.0005
Weng, Nutrients, 2013 - ARIC	9913	2853	1.18	[1.12, 1.25]	<0.00001	67%	0.0004
Winkelmayer, JAMA, 2005 - NHS	61091	19541	1.16	[1.10, 1.23]	<0.00001	68%	0.0003
Winkelmayer, JAMA, 2005 - NHSII	94503	13536	1.16	[1.10, 1.23]	<0.00001	68%	0.0003
**Fruit**							
All included:	281,120	148,928	0.81	[0.73, 0.89]	<0.0001	88%	<0.00001
Auerbach, Prev med, 2017 - WHI	80539	46202	0.77	[0.68, 0.87]	<0.0001	87%	<0.00001
Borgi, Hypertension, 2016 - HPFS	20010	16752	0.79	[0.70, 0.89]	<0.0001	89%	<0.00001
Borgi, Hypertension, 2016 - NHS	39164	35375	0.78	[0.69, 0.88]	<0.0001	89%	<0.00001
Borgi, Hypertension, 2016 - NHS II	63885	25246	0.79	[0.71, 0.88]	<0.0001	89%	<0.00001
Kim, J Acad Nutr Diet, 2017 - KoGES (men)	2085	606	0.84	[0.77, 0.93]	0.0005	86%	<0.00001
Kim, J Acad Nutr Diet, 2017 - KoGES (women)	2172	552	0.87	[0.80, 0.94]	0.0009	80%	<0.00001
Koochakpoor, Nutr Res, 2018 - TLGS	1284	640	0.80	[0.72, 0.89]	<0.0001	89%	<0.00001
Nun˜ez-Cordoba, Eur J Clin Nutr, 2009 - SUN	8594	426	0.80	[0.72, 0.89]	<0.0001	89%	<0.00001
Psaltopoulou, Am J Clin Nutr, 2004 - EPIC	20343	5424	0.82	[0.74, 0.91]	0.0002	88%	<0.00001
Steffen, Am J Clin Nutr, 2005 - CARDIA	4304	997	0.81	[0.73, 0.90]	0.0001	88%	<0.00001
Tsubota-Utsugi, J Hum Hypertens, 2011 - Ohasama	745	222	0.82	[0.74, 0.91]	0.0001	88%	<0.00001
Wang, Am J Hypertens, 2012 - WHS	28082	13633	0.78	[0.69, 0.88]	<0.0001	89%	<0.00001
Weng, Nutrients, 2013 - ARIC	9913	2853	0.78	[0.70, 0.87]	<0.00001	89%	<0.00001
**Yogurt**							
All included:	235705	97783	0.96	[0.86, 0.96]	0.0007	54%	0.03
Alonso, Eur J Clin Nutr, 2009 - ARIC	8208	2399	0.90	[0.85, 0.95]	0.0002	54%	0.03
Buendia, J Hypertens, 2018 - HPFS	30512	14166	0.90	[0.85, 0.95]	0.0002	53%	0.04
Buendia, J Hypertens, 2018 - NHS	69298	41934	0.92	[0.86, 0.98]	0.007	53%	0.04
Buendia, J Hypertens, 2018 - NHSII	84368	26282	0.91	[0.85, 0.97]	0.006	59%	0.02
Engberink, J Nutr, 2009 - MORGEN	3454	713	0.91	[0.86, 0.97]	0.002	58%	0.02
**Kim, Brit J Nutr, 2017 - KoGES**	4335	1556	0.92	[0.88, 0.96]	0.0003	**30%**	**0.19**
Steffen, Am J Clin Nutr, 2005 - CARDIA	4304	997	0.91	[0.86, 0.97]	0.002	60%	0.02
Wang, Brit J Nutr, 2015 - FHS	28886	8710	0.90	[0.84, 0.96]	0.002	53%	0.04
Wang, Hypertension, 2008 - WHS	2340	1026	0.90	[0.85, 0.96]	0.0008	58%	0.02

Each study was removed independently and the pooled estimate recalculated. The red and blue lines represent the original pooled risk estimate with all studies included. Inter-study heterogeneity was tested using the Cochran Q statistic (Chi^2) at a significance level of $p<0.10$, and quantified by the I^2 statistic. An I^2 value ⩾ 50% is considered to indicate substantial heterogeneity. All results are presented as Relative Risks (RR) with 95% Confidence Intervals.

Table S6. **GRADE assessment.**

Quality assessment									Effect	Quality
No. of comparisons	Design	Risk of bias	Inconsistency	Indirectness	Imprecision	Publication bias	Other considerations	Study event rates (%)	Relative Risk [95% CI]	Importance
SSBs intake on incident hypertension (follow-up median 10.0 years)										
13 [24, 26-29, 32, 38, 44-46]	Observational studies	No serious	Serious*	No serious	No serious	Detected†	Dose-response gradient‡	28%	RR 1.17 [1.11, 1.23]	⊕⊕⊖⊖ Low *,†, ‡
Fruit intake on incident hypertension (follow-up median 9.0 years)										
13 [25, 30, 31, 33, 34, 36, 40, 46, 48, 49]	Observational studies	No serious	Serious§	No serious	No serious	Detected ‖	Dose-response gradient#	53%	RR 0.81 [0.73, 0.89]	⊕⊕⊖⊖ Low §, ‖, #
Yogurt intake on incident hypertension (follow-up median 14.6 years)										
9 [33, 35, 37, 41-43, 47]	Observational studies	No serious	Serious**	No serious	Serious††	Not detected‡‡	Dose-response gradient§§	41%	RR 0.91 [0.86, 0.96]	⊕⊕⊖⊖ Low **,††,‡‡,§§
Dairy desserts intake on incident hypertension (follow-up median 10.0 years)										
3 [33, 35, 42]	Observational studies	No serious	No serious	No serious	Serious††	Not detected‡‡	None	29%	RR 0.85 [0.76, 0.95]	⊕⊖⊖⊖ Very low ††,‡‡
100% Fruit juice intake on incident hypertension (follow-up median 13.9 years)										
2 [28, 40]	Observational studies	No serious	Serious‖‖	No serious	No serious##	Not detected‡‡	Dose-response gradient***	56%	RR 0.95 [0.85, 1.07]	⊕⊕⊖⊖ Low ‡‡,‖‖,##,***
Fruit drinks intake on incident hypertension (follow-up 3.6 years)										
1 [29]	Observational study	No serious	No serious	Serious†††,‡‡‡	Serious§§§	Not detected‡‡	None	11%	RR 1.27 [0.43, 3.75]	⊕⊖⊖⊖ Very low ‡‡,†††,‡‡‡,§§§
Sweet snacks intake on incident hypertension (follow-up 3.6 years)										
1 [39]	Observational study	No serious	No serious	Serious†††,‡‡‡	Serious‖‖‖	Not detected‡‡	None	11%	RR 2.00 [0.84, 4.76]	⊕⊖⊖⊖ Very low ‡‡,***,†††,‖‖‖

* Downgrade for serious inconsistency, as there was evidence of substantial inter-study heterogeneity (I^2=66%, p=0.0004)
† There was evidence of funnel plot asymmetry via visual inspection and both the Egger (p=0.02) and Begg test were significant (p=0.04). Adjustment for funnel plot asymmetry by the recalculation of the pooled estimate by inputting missing studies using the Duvall and Tweedie trim and fill method did not alter the significance of the relationship, with only limited attenuation of the summary estimate (RR=1.12 [95% CI, 1.05-1.19]).
‡ Upgrade for dose-response gradient, as there was a significant harmful dose-response relationship between SSBs intake and hypertension with evidence for non-linearity (p=0.02).
§ Downgrade for serious inconsistency, as there was evidence of substantial inter-study heterogeneity (I^2=88%, p<0.00001).
‖ There was evidence of funnel plot asymmetry as the Begg test was significant (p=0.09), although the Egger test was not significant (p=0.70). The Duvall and Tweedie trim and fill method did not perform any trimming and the pooled estimate did not change.
Upgrade for dose-response gradient, as there was a significant protective and linear dose-response relationship between fruit intake and hypertension.
** Downgrade for serious inconsistency, as there was evidence of substantial inter-study heterogeneity (I^2=54%, p=0.03)
†† Downgrade for serious imprecision, as the upper CI bound crosses the clinically important protection threshold of RR=0.9.
‡‡ Bias cannot be excluded since we were unable to test for funnel plot asymmetry due to lack of power (<10 cohorts included in the analysis).
§§ Upgrade for dose-response gradient, as there was a significant protective dose-response relationship between yogurt intake and hypertension with evidence for non-linearity (p=0.02).
‖‖ Downgrade for serious inconsistency, as there was evidence of substantial inter-study heterogeneity (I^2=85%, p=0.01).
Although pairwise meta-analysis showed serious imprecision, this imprecision was explained by non-linear dose-response analysis.
*** Upgrade for dose-response gradient, as there was a significant U-shaped dose-response relationship between 100% fruit juice intake and hypertension (P-value for non-linearity=0.001).
††† Downgrade for serious indirectness due to limited number of cohort comparisons in specific groups which may not be generalizable to the general population.
‡‡‡ Downgrade for serious indirectness, as only number of cases of metabolic syndrome was reported.
§§§ Downgrade as the sample sizes were very small (n=424) and the 95% CI were very large (0.43, 3.75) containing evidence of both clinically important protection (RR<0.9) and harm (RR>1.1).
‖‖‖ Downgrade as the sample size was very small (n=439) and the 95% CI were very large (0.84, 4.76) containing evidence of both clinically important protection (RR<0.9) and harm (RR>1.1)

Figure S1. Forest plot – Pairwise meta-analysis of SSBs intake and incident hypertension.

Author, year - Cohort	N, Participants	N, Cases	Weight	Risk Ratio [95% CI] for Incident Hypertension, Random
Barrio-Lopez, Brit J Nutr, 2013 - SUN	8157	1464	5.4%	1.53 [1.25, 1.85]
Cohen, J Gen Intern Med, 2012 - HPFS	37360	13439	13.6%	1.06 [0.99, 1.14]
Cohen, J Gen Intern Med, 2012 - NHS	88540	42022	16.0%	1.12 [1.08, 1.17]
Cohen, J Gen Intern Med, 2012 - NHSII	97991	21873	15.1%	1.17 [1.11, 1.23]
Dhingra, Circulation, 2007 - FOC	2803	1377	4.6%	1.14 [0.92, 1.42]
Duffrey, Am J Clin Nutr, 2010 - CARDIA	2639	609	15.1%	1.06 [1.01, 1.12]
Kang, Brit J Nutr, 2017 - KoGES	4591	1309	3.3%	1.55 [1.18, 2.04]
Kwak, Eur J Nutr, 2018 - KoGES	5775	1175	6.3%	1.21 [1.02, 1.45]
Mirmiran, Nutr Metab, 2015 - TLGS	424	47	0.8%	2.02 [1.13, 3.60]
Sayon-Orea, Clin Nutr, 2015 - SUN	13843	1308	5.0%	1.34 [1.09, 1.65]
Weng, Nutrients, 2013 - ARIC	9913	2853	8.8%	1.03 [0.90, 1.17]
Winkelmayer, JAMA, 2005 - NHS	61091	19541	1.8%	1.44 [0.98, 2.11]
Winkelmayer, JAMA, 2005 - NHSII	94503	13536	4.1%	1.28 [1.01, 1.62]
**Total (95% CI)**	**427630**	**120553**	**100.0%**	**1.17 [1.11, 1.23]**

Heterogeneity: $Tau^2 = 0.00$; $Chi^2 = 35.66$, $df = 12$ ($P = 0.0004$); $I^2 = 66\%$
Test for overall effect: $Z = 5.55$ ($P < 0.00001$)

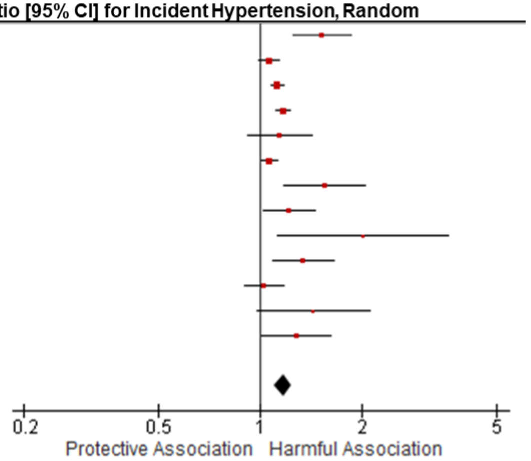

The black diamond represents the pooled risk estimate. Inter-study heterogeneity was tested using the Cochran Q statistic (Chi^2) at a significance level of $p<0.10$, and quantified by the I^2 statistic. An I^2 value ⩾ 50% is considered to indicate substantial heterogeneity. All results are presented as Relative Risks (RR) with 95% Confidence Intervals

Figure S2. Forest plot – Pairwise meta-analysis of fruit intake and incident hypertension.

Author, year - Cohort	N, Participants	N, Cases	Weight	Risk Ratio [95% CI] for Incident Hypertension, Random
Auerbach, Prev med, 2017 - WHI	80539	46202	11.4%	1.00 [0.97, 1.04]
Borgi, Hypertension, 2016 - HPFS	20010	16752	10.6%	0.89 [0.81, 0.97]
Borgi, Hypertension, 2016 - NHS	39164	35375	10.8%	0.95 [0.88, 1.03]
Borgi, Hypertension, 2016 - NHS II	63885	25246	10.0%	0.91 [0.81, 1.02]
Kim, J Acad Nutr Diet, 2017 - KoGES (men)	2085	606	5.4%	0.44 [0.32, 0.61]
Kim, J Acad Nutr Diet, 2017 - KoGES (women)	2172	552	5.4%	0.33 [0.24, 0.45]
Koochakpoor, Nutr Res, 2018 - TLGS	1284	640	5.0%	0.89 [0.63, 1.26]
Nun˜ez-Cordoba, Eur J Clin Nutr, 2009 - SUN	8594	426	4.7%	0.85 [0.59, 1.22]
Psaltopoulou, Am J Clin Nutr, 2004 - EPIC	20343	5424	5.7%	0.61 [0.45, 0.83]
Steffen, Am J Clin Nutr, 2005 - CARDIA	4304	997	7.4%	0.75 [0.60, 0.94]
Tsubota-Utsugi, J Hum Hypertens, 2011 - Ohasama	745	222	2.9%	0.49 [0.29, 0.81]
Wang, Am J Hypertens, 2012 - WHS	28082	13633	10.8%	0.95 [0.88, 1.03]
Weng, Nutrients, 2013 - ARIC	9913	2853	9.7%	1.06 [0.93, 1.21]
**Total (95% CI)**	**281120**	**148928**	**100.0%**	**0.81 [0.73, 0.89]**

Heterogeneity: Tau² = 0.02; Chi² = 99.26, df = 12 (P < 0.00001); I² = 88%
Test for overall effect: Z = 4.15 (P < 0.0001)

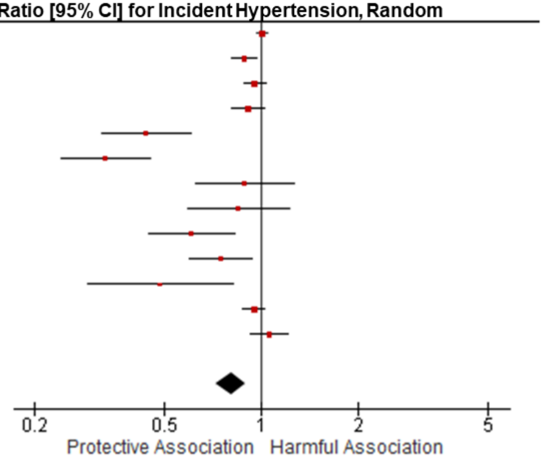

The black diamond represents the pooled risk estimate. Inter-study heterogeneity was tested using the Cochran Q statistic (Chi^2) at a significance level of $p<0.10$, and quantified by the I^2 statistic. An I^2 value ⩾ 50% is considered to indicate substantial heterogeneity. All results are presented as Relative Risks (RR) with 95% Confidence Intervals. Mirmiran et al. only reported cases of metabolic syndrome.

Figure S3. Forest plot – Pairwise meta-analysis of yogurt intake and incident hypertension.

Author, year - Cohort	N, Participants	N, Cases	Weight	Risk Ratio [95% CI] for Incident Hypertension, Random
Alonso, Eur J Clin Nutr, 2009 - ARIC	8208	2399	4.0%	1.11 [0.86, 1.43]
Buendia, J Hypertens, 2018 - HPFS	30512	14166	10.8%	1.01 [0.89, 1.15]
Buendia, J Hypertens, 2018 - NHS	69298	41934	17.3%	0.87 [0.81, 0.93]
Buendia, J Hypertens, 2018 - NHSII	84368	26282	15.9%	0.89 [0.82, 0.97]
Engberink, J Nutr, 2009 - MORGEN	3454	713	6.9%	0.84 [0.71, 1.01]
Kim, Brit J Nutr, 2017 - KoGES	4335	1556	6.6%	0.71 [0.59, 0.85]
Steffen, Am J Clin Nutr, 2005 - CARDIA	4304	997	7.8%	0.88 [0.75, 1.04]
Wang, Brit J Nutr, 2015 - FHS	28886	8710	20.5%	0.94 [0.90, 0.99]
Wang, Hypertension, 2008 - WHS	2340	1026	10.2%	0.97 [0.85, 1.11]
**Total (95% CI)**	**235705**	**97783**	**100.0%**	**0.91 [0.86, 0.96]**

Heterogeneity: $Tau^2 = 0.00$; $Chi^2 = 17.49$, df = 8 (P = 0.03); $I^2 = 54\%$
Test for overall effect: Z = 3.39 (P = 0.0007)

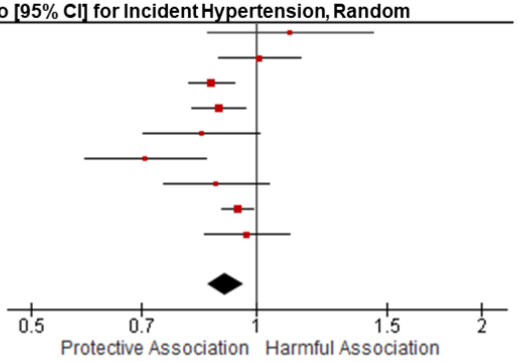

The black diamond represents the pooled risk estimate. Inter-study heterogeneity was tested using the Cochran Q statistic (Chi^2) at a significance level of p<0.10, and quantified by the I^2 statistic. An I^2 value ⩾ 50% is considered to indicate substantial heterogeneity. All results are presented as Relative Risks (RR) with 95% Confidence Intervals.

Figure S4. Forest plot – Pairwise meta-analysis of dairy desserts intake and incident hypertension.

Author, year - Cohort	N, Participants	N, Cases	Weight	Risk Ratio [95% CI] for Incident Hypertension, Random
Alonso, Eur J Clin Nutr, 2009 - ARIC	8208	2399	36.8%	0.88 [0.74, 1.05]
Steffen, Am J Clin Nutr, 2005 - CARDIA	4304	997	24.7%	0.74 [0.60, 0.92]
Wang, Hypertension, 2008 - WHS	28886	8710	38.5%	0.90 [0.76, 1.07]
**Total (95% CI)**	**41398**	**12106**	**100.0%**	**0.85 [0.76, 0.95]**

Heterogeneity: Tau² = 0.00; Chi² = 2.11, df = 2 (P = 0.35); I² = 5%
Test for overall effect: Z = 2.91 (P = 0.004)

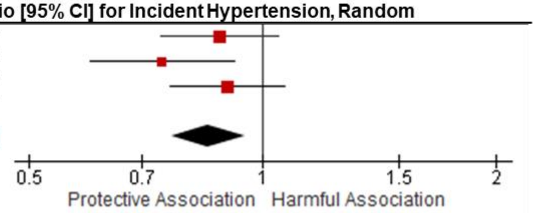

The black diamond represents the pooled risk estimate. Inter-study heterogeneity was tested using the Cochran Q statistic (Chi^2) at a significance level of $p<0.10$, and quantified by the I^2 statistic. An I^2 value ⩾ 50% is considered to indicate substantial heterogeneity. All results are presented as Relative Risks (RR) with 95% Confidence Intervals.

Figure S5. Forest plot – Pairwise meta-analysis of 100% fruit juice intake and incident hypertension.

Author, year - Cohort	N, Participants	N, Cases	Weight	Risk Ratio [95% CI] for Incident Hypertension, Random
Auerbach, Prev med, 2017 - WHI	80539	46202	55.4%	1.00 [0.97, 1.04]
Duffrey, Am J Clin Nutr, 2010 - CARDIA	2639	609	44.6%	0.89 [0.82, 0.97]
**Total (95% CI)**	**83178**	**46811**	**100.0%**	**0.95 [0.85, 1.07]**

Heterogeneity: $Tau^2 = 0.01$; $Chi^2 = 6.55$, df = 1 (P = 0.01); $I^2 = 85\%$
Test for overall effect: Z = 0.82 (P = 0.41)

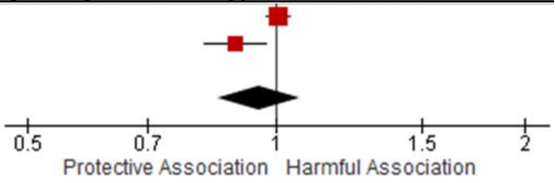

The black diamond represents the pooled risk estimate. Inter-study heterogeneity was tested using the Cochran Q statistic (Chi^2) at a significance level of p<0.10, and quantified by the I^2 statistic. An I^2 value ⩾ 50% is considered to indicate substantial heterogeneity. All results are presented as Relative Risks (RR) with 95% Confidence Intervals.

Figure S6. Forest plot – Pairwise meta-analysis of fruit drinks intake and incident hypertension.

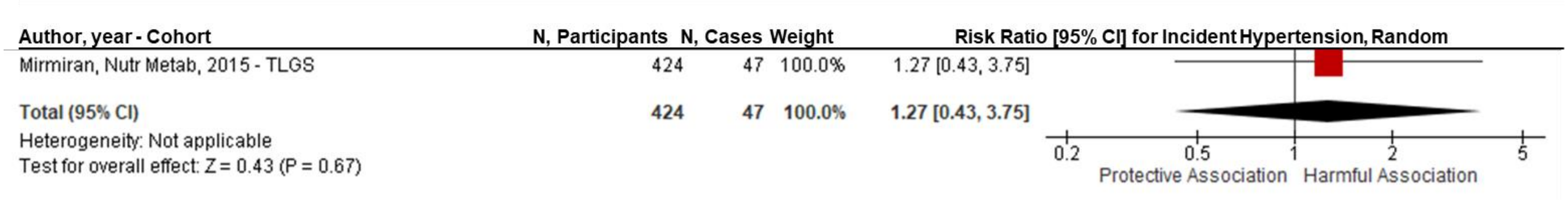

The black diamond represents the pooled risk estimate. Inter-study heterogeneity was tested using the Cochran Q statistic (Chi^2) at a significance level of $p<0.10$, and quantified by the I^2 statistic. An I^2 value ⩾ 50% is considered to indicate substantial heterogeneity. All results are presented as Relative Risks (RR) with 95% Confidence Intervals. *Study only reported cases of metabolic syndrome.

Figure S7. Forest plot – Pairwise meta-analysis of sweet Snacks intake and incident hypertension.

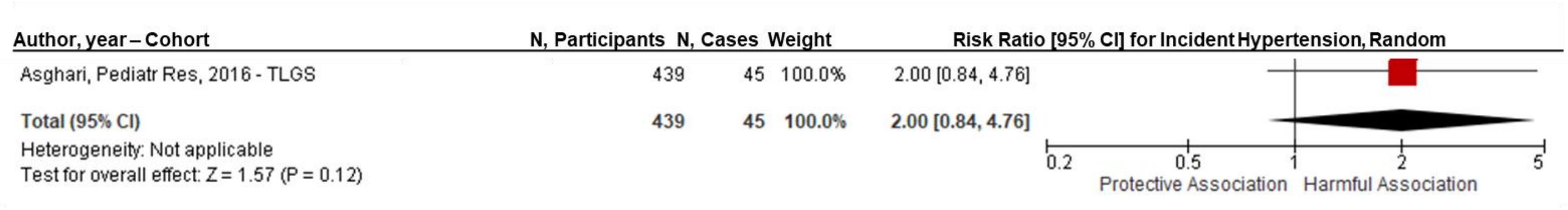

The black diamond represents the pooled risk estimate. Inter-study heterogeneity was tested using the Cochran Q statistic (Chi^2) at a significance level of $p<0.10$, and quantified by the I^2 statistic. An I^2 value ⩾ 50% is considered to indicate substantial heterogeneity. All results are presented as Relative Risks (RR) with 95% Confidence Intervals. *Study only reported cases of metabolic syndrome.

Figure S8. Dose-response relation between sources of fructose-containing sugars and incident hypertension with study-specific data points.

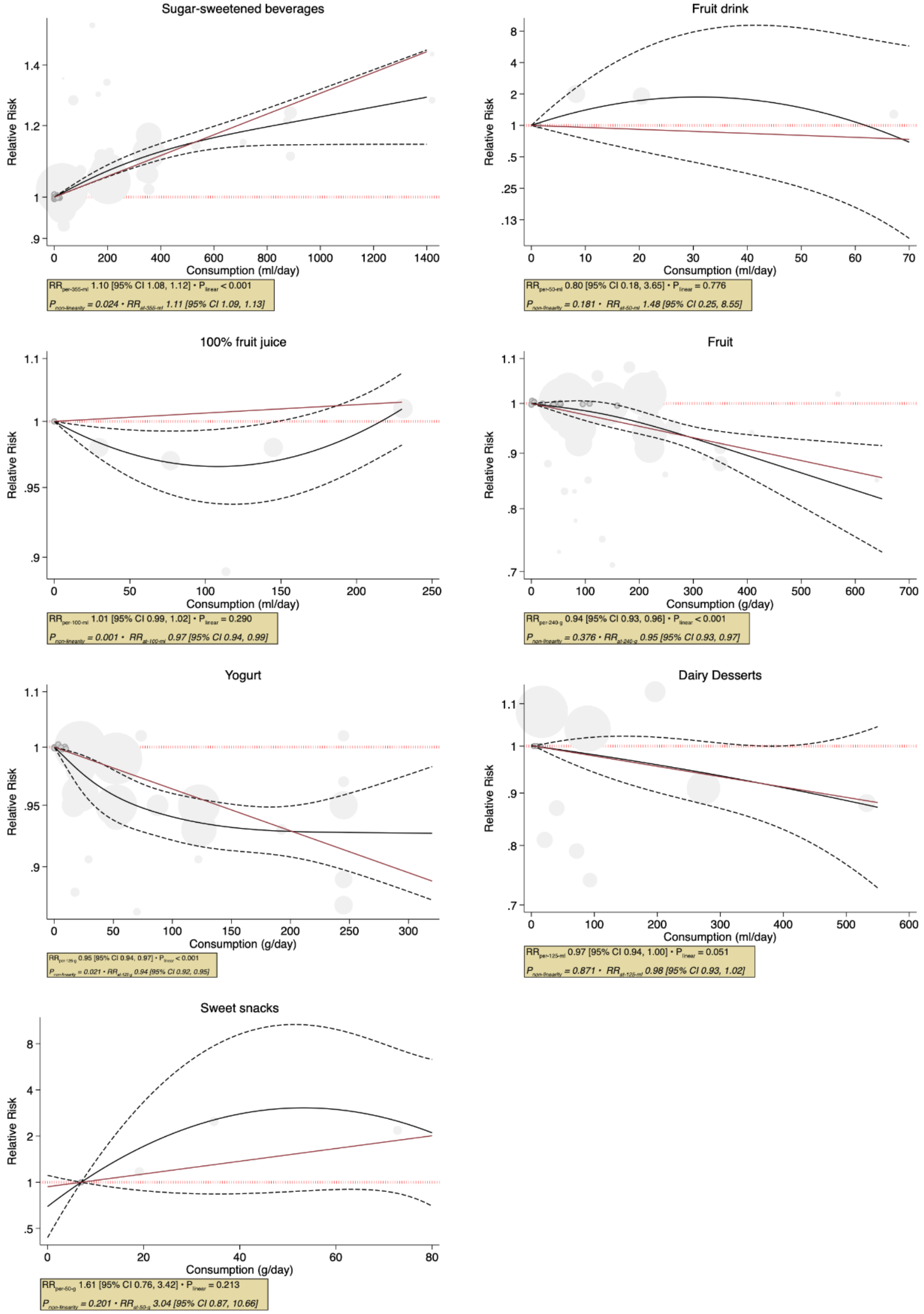

Dose-response relationship between intake of SSBs, fruit, 100% fruit juice, yogurt, fruit drink, dairy desserts, and sweet snacks with risk of hypertension. Red line represents the linear and black lines represent the non-linear models, respectively. Dotted lines represent 95% confidence intervals of the non-linear model. The light gray circles represent the relative risk-point estimates for the different doses from each study; the size of the circle is related to inverse of the variance. The smaller gray circles with dark gray outline represent the baseline dose category for each separate study; random-noise has been added in the graphic display for these baseline circles to show them separately.

Figure S9. Subgroup analyses of SSBs intake and incident hypertension.

Subgroup	Level	Cohort Comparisons	Subjects	Relative Risk [95% CI] on incident Hypertension: Within subgroups	Relative Risk [95% CI] on incident Hypertension: Between subgroups	Residual I^2	P-Interaction
Total	-	13	427,630	1.19 [1.09, 1.30]	-	66%	-
Sex	Females (1)	4	342,125	1.16 [0.97, 1.40]	1 vs. 2: 0.99 [0.71, 1.40], p=0.96	70%	0.281
	Males (2)	1	37,360	1.17 [0.85, 1.60]	1 vs. 3: 0.94 [0.75, 1.17], p=0.52		
	Both (3)	8	48,145	1.23 [1.07, 1.42]	2 vs.3: 1.06 [0.77, 1.46], p=0.70		
Follow-up	<10 years	6	40,915	1.24 [1.06, 1.44]	1.07 [0.90, 1.29], p=0.40	66%	0.07
	≥10 years	7	386,715	1.17 [1.03, 1.32]			
NOS	<6	0					
	≥6	13	427,630	1.19 [1.09, 1.31]	-	66%	-
Age	<36.4 years (median)	6	292,254	1.19 [1.03, 1.37]	0.97 [0.81, 1.17], p=0.74	64%	0.03
	≥36.4 years (median)	7	135,376	1.21 [1.05, 1.39]			
Funding	Agency	13	427,630	1.19 [1.09, 1.30]	-	66%	-
	Industry	0					
	Both	0					

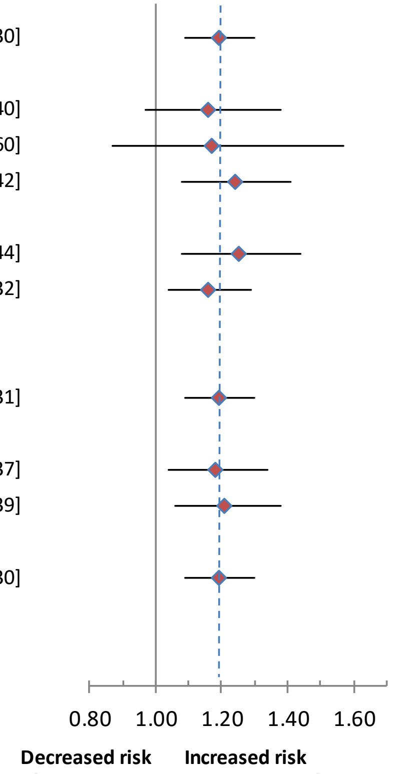

RR, relative risk; NOS, Newcastle-Ottawa Scale. Point estimates for each subgroup level (diamonds) are the pooled effect estimates. The dashed line represents the pooled effect estimate for the overall (total) analysis. The residual I^2 value indicates the inter-study heterogeneity unexplained by the subgroup.

Figure S10. Subgroup analyses of fruit intake and incident hypertension.

Subgroup	Level	Cohort Comparisons	Subjects	Relative Risk [95% CI] on incident Hypertension: Within subgroups	Relative Risk [95% CI] on incident Hypertension: Between subgroups	Residual I^2	P-Interaction
Total	-	13	281,120	0.76 [0.62, 0.94]	-	88%	-
Sex	Females (1)	5	213,842	0.80 [0.79, 1.14]	1 vs. 2: 1.21 [0.60, 2.45], p=0.56	89%	<0.001
	Males (2)	2	22,095	0.65 [0.36, 1.19]	1 vs. 3: 1.02 [0.61, 1.71], p=0.93		
	Both (3)	6	45,183	0.77 [0.54, 1.10]	2 vs. 3: 1.18 [0.59, 2.38], p=0.60		
Follow-up	<10 years	7	124,391	0.66 [0.49, 0.87]	0.74 [0.49, 1.10], p=0.12	89%	<0.001
	≥10 years	6	156,729	0.89 [0.67, 1.18]			
NOS	<6	1	28,082	0.95 [0.45, 2.03]	1.27 [0.58, 2.81], p=0.52	91%	0.10
	≥6	12	253,038	0.75 [0.59, 0.94]			
Age	<53 years (median)	6	117,976	0.81 [0.58, 1.13]	1.12 [0.71, 1.75], p=0.60	88%	<0.001
	≥53 years (median)	7	163,144	0.72 [0.54, 0.98]			
Funding	Agency	12	253,038	0.75 [0.59, 0.94]	1 vs. 3: 0.79 [0.36, 1.73], p=0.52	91%	0.10
	Industry	0					
	Both	1	28,082	0.95 [0.45, 2.03]			

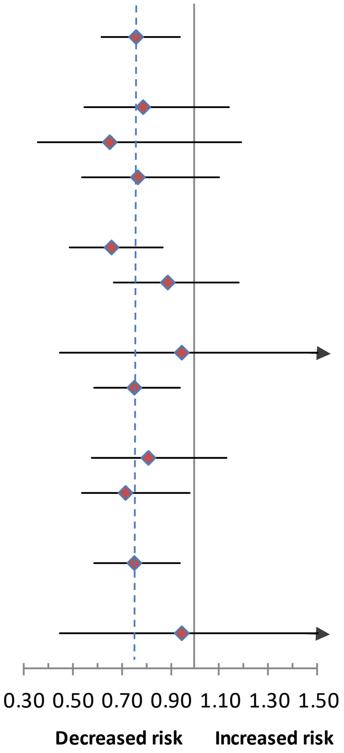

RR, relative risk; NOS, Newcastle-Ottawa Scale. Point estimates for each subgroup level (diamonds) are the pooled effect estimates. The dashed line represents the pooled effect estimate for the overall (total) analysis. The residual I^2 value indicates the inter-study heterogeneity unexplained by the subgroup.

Figure S11. Funnel plot of natural logarithm relative risk (RR) for incident hypertension comparing the highest and lowest quantiles of SSBs intake.

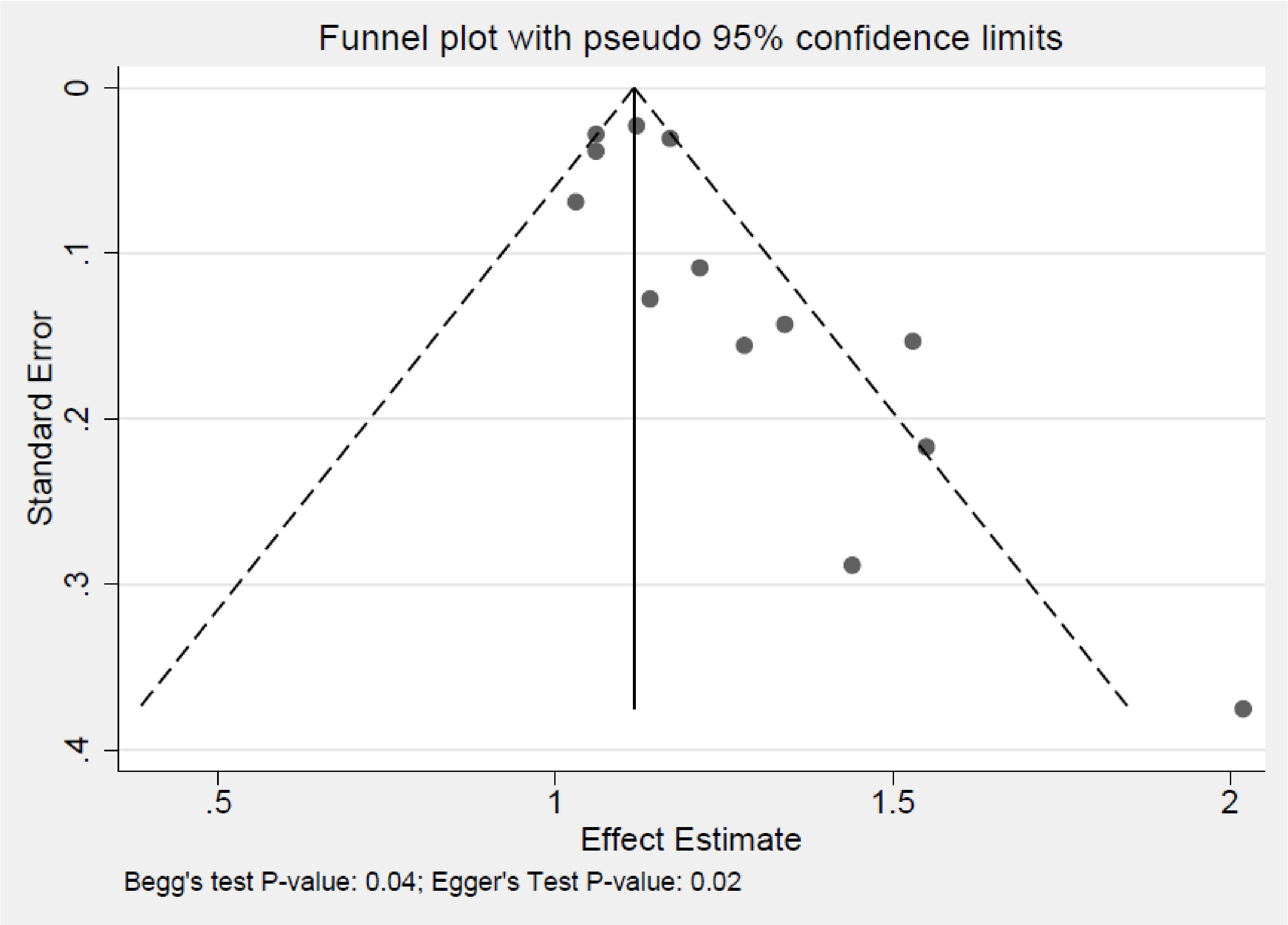

The vertical line represents the pooled effect estimate expressed as natural logarithm RR. Dashed lines represent pseudo-95% confidence intervals (CI). The circles represent risk effects for each cohort, and the horizontal lines represent standard errors of the effect estimate.

Figure S12. Trim and fill funnel plot of natural logarithm relative risk (RR) for incident hypertension comparing the highest and lowest quantiles of SSBs intake.

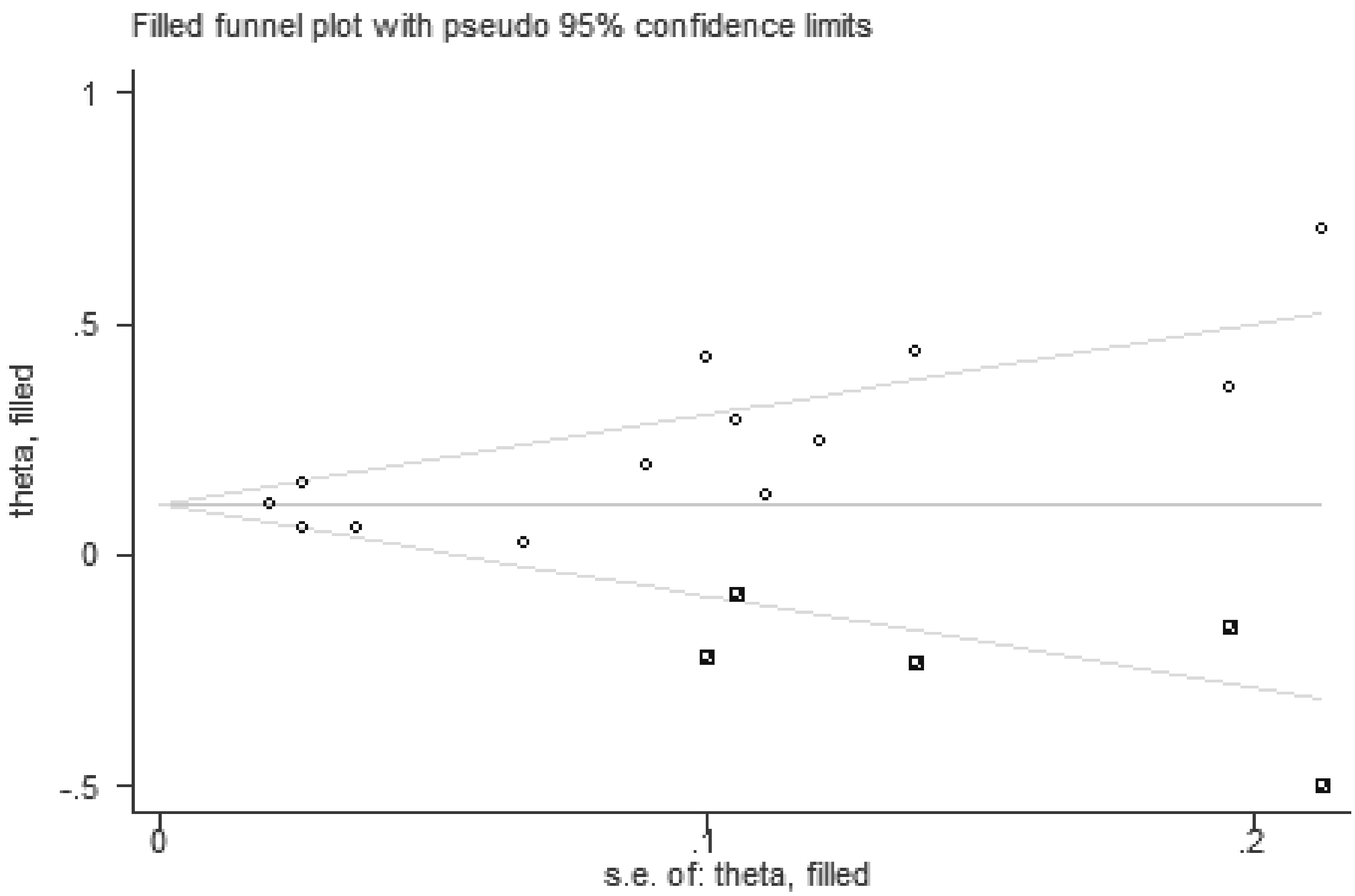

The horizontal line represents the pooled effect estimate expressed as natural logarithm RR. Diagonal lines represent pseudo-95% confidence intervals (CI). The circles represent risk effects for each cohort, and the squares represent filled data points. The horizontal axis represents standard errors of the effect estimate. Adjustment for funnel plot asymmetry by the recalculation of the pooled estimate by inputting missing cohort studies using the Duvall and Tweedie trim and fill method did not alter the significance of the relationship with only limited attenuation of the summary estimate (RR=1.12 [95% CI, 1.05, 1.19] versus original RR=1.17 [95% CI, 1.11, 1.23]).

Figure S13. Funnel plot of natural logarithm relative risk (RR) for incident hypertension comparing the highest and lowest quantiles of fruit intake.

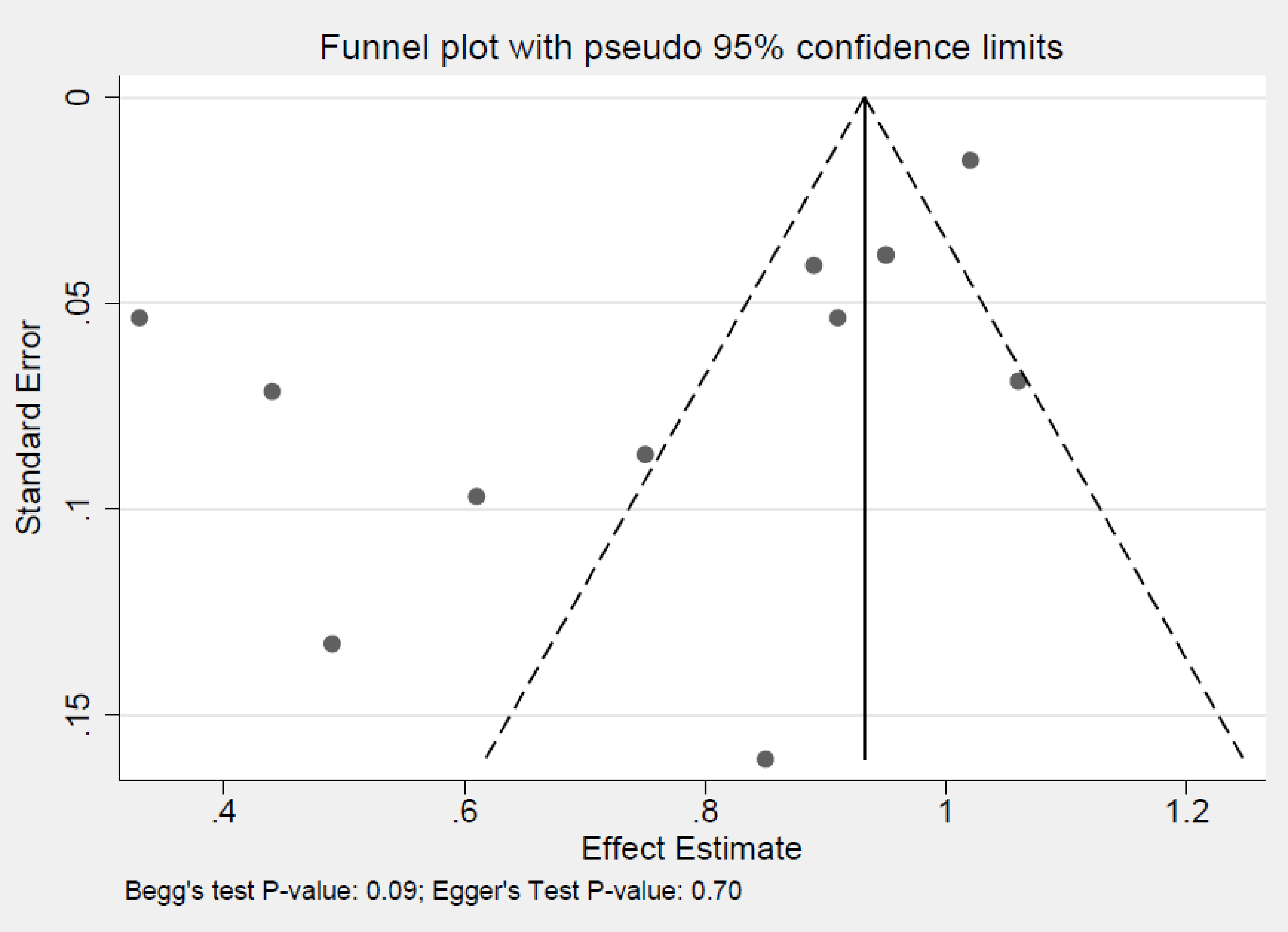

The vertical line represents the pooled effect estimate expressed as natural logarithm RR. Dashed lines represent pseudo-95% confidence intervals (CI). The circles represent risk effects for each cohort, and the horizontal lines represent standard errors of the effect estimate.

Supplemental References:

1. Barrio-Lopez MT, Martinez-Gonzalez MA, Fernandez-Montero A, Beunza JJ, Zazpe I, Bes-Rastrollo M. Prospective study of changes in sugar-sweetened beverage consumption and the incidence of the metabolic syndrome and its components: The sun cohort. *British Journal of Nutrition*. 2013;110:1722-1731.
2. Cohen LC, G. Forman, J. Association of sweetened beverage intake with incident hypertension. *Journal of General Internal Medicine*. 2012;27:1127-1134.
3. Dhingra R, Sullivan L, Jacques PF, Wang TJ, Fox CS, Meigs JB, D'Agostino RB, Gaziano JM, Vasan RS. Soft drink consumption and risk of developing cardiometabolic risk factors and the metabolic syndrome in middle-aged adults in the community.[erratum appears in circulation. 2007 dec 4;116(23):E557]. *Circulation*. 2007;116:480-488.
4. Duffey KJG-L, P. Steffen, L. M. Jacobs Jr, D. R. Popkin, B. M. Drinking caloric beverages increases the risk of adverse cardiometabolic outcomes in the coronary artery risk development in young adults (cardia) study. *American Journal of Clinical Nutrition*. 2010;92:954-959.
5. Kang Y, Kim J. Soft drink consumption is associated with increased incidence of the metabolic syndrome only in women. *British Journal of Nutrition*. 2017;117:315-324.
6. Kwak JH, Jo G, Chung HK, Shin MJ. Association between sugar-sweetened beverage consumption and incident hypertension in korean adults: A prospective study. *European Journal of Nutrition*. 2018;25:25.
7. Mirmiran PY, E. Asghari, G. Hosseinpour-Niazi, S. Azizi, F. Consumption of sugar sweetened beverage is associated with incidence of metabolic syndrome in tehranian children and adolescents. *Nutrition and Metabolism*. 2015;12.
8. Sayon-Orea C, Martinez-Gonzalez MA, Gea A, Alonso A, Pimenta AM, Bes-Rastrollo M. Baseline consumption and changes in sugar-sweetened beverage consumption and the incidence of hypertension: The sun project. *Clinical Nutrition*. 2015;34:1133-1140.
9. Weng L-C, Steffen LM, Szklo M, Nettleton J, Chambless L, Folsom AR. A diet pattern with more dairy and nuts, but less meat is related to lower risk of developing hypertension in middle-aged adults: The atherosclerosis risk in communities (aric) study. *Nutrients*. 2013;5:1719-1733.
10. Winkelmayer WC, Stampfer MJ, Willett WC, Curhan GC. Habitual caffeine intake and the risk of hypertension in women. *Jama*. 2005;294:2330-2335.
11. Borgi L, Muraki I, Satija A, Willett WC, Rimm EB, Forman JP. Fruit and vegetable consumption and the incidence of hypertension in three prospective cohort studies. *Hypertension*. 2016;67:288-293.
12. Kim J, Kim J. Association between fruit and vegetable consumption and risk of hypertension in middle-aged and older korean adults. *J Acad Nutr Diet*. 2017;04:04.
13. Auerbach BJ, Littman AJ, Tinker L, Larson J, Krieger J, Young B, Neuhouser M. Associations of 100% fruit juice versus whole fruit with hypertension and diabetes risk in postmenopausal women: Results from the women's health initiative. *Preventive medicine*. 2017;105:212-218.
14. Koochakpoor GM, P. Daneshpour, M. S. Hosseini-Esfahani, F. Sedaghati-Khayat, B. Hosseini, S. A. Azizi, F. Dietary factors influence the association of cyclin d2 polymorphism rs11063069 with the risk of metabolic syndrome. *Nutrition Research*. 2018;52:48-56.

15. Nunez-Cordoba JM, Alonso A, Beunza JJ, Palma S, Gomez-Gracia E, Martinez-Gonzalez MA. Role of vegetables and fruits in mediterranean diets to prevent hypertension. *European Journal of Clinical Nutrition*. 2009;63:605-612.
16. Psaltopoulou T, Naska A, Orfanos P, Trichopoulos D, Mountokalakis T, Trichopoulou A. Olive oil, the mediterranean diet, and arterial blood pressure: The greek european prospective investigation into cancer and nutrition (epic) study.[erratum appears in am j clin nutr. 2005 may;81(5):1181]. *American Journal of Clinical Nutrition*. 2004;80:1012-1018.
17. Steffen LMK, C. H. Yu, X. Pereira, M. A. Slattery, M. L. Van Horn, L. Gross, M. D. Jacobs Jr, D. R. Associations of plant food, dairy product, and meat intakes with 15-y incidence of elevated blood pressure in young black and white adults: The coronary artery risk development in young adults (cardia) study. *The American journal of clinical nutrition*. 2005;82:1169-1177; quiz 1363-1364.
18. Tsubota-Utsugi MO, T. Kikuya, M. Metoki, H. Kurimoto, A. Suzuki, K. Fukushima, N. Hara, A. Asayama, K. Satoh, H. Tsubono, Y. Imai, Y. High fruit intake is associated with a lower risk of future hypertension determined by home blood pressure measurement: The ohasama study. *Journal of Human Hypertension*. 2011;25:164-171.
19. Wang LM, J. E. Gaziano, J. M. Buring, J. E. Sesso, H. D. Fruit and vegetable intake and the risk of hypertension in middle-aged and older women. *American Journal of Hypertension*. 2012;25:180-189.
20. Wang L, Manson JE, Buring JE, Lee I-M, Sesso HD. Dietary intake of dairy products, calcium, and vitamin d and the risk of hypertension in middle-aged and older women. *Hypertension*. 2008;51:1073-1079.
21. Buendia JR, Li Y, Hu FB, Cabral HJ, Bradlee ML, Quatromoni PA, Singer MR, Curhan GC, Moore LL. Long-term yogurt consumption and risk of incident hypertension in adults. *Journal of Hypertension*. 2018;36:1671-1679.
22. Alonso A, Steffen LM, Folsom AR. Dairy intake and changes in blood pressure over 9 years: The aric study. *European journal of clinical nutrition*. 2009;63:1272.
23. Engberink MF, Geleijnse JM, de Jong N, Smit HA, Kok FJ, Verschuren WM. Dairy intake, blood pressure, and incident hypertension in a general dutch population. *The Journal of nutrition*. 2009;139:582-587.
24. Kim DK, J. Dairy consumption is associated with a lower incidence of the metabolic syndrome in middle-aged and older korean adults: The korean genome and epidemiology study (koges). *British Journal of Nutrition*. 2017;117:148-160.
25. Wang H, Fox CS, Troy LM, McKeown NM, Jacques PF. Longitudinal association of dairy consumption with the changes in blood pressure and the risk of incident hypertension: The framingham heart study. *British Journal of Nutrition*. 2015;114:1887-1899.
26. Asghari G, Yuzbashian E, Mirmiran P, Bahadoran Z, Azizi F. Prediction of metabolic syndrome by a high intake of energy-dense nutrient-poor snacks in iranian children and adolescents. *Pediatric research*. 2016;79:697.
